# *Vibrio cholerae* ensures function of host proteins required for virulence through consumption of luminal methionine sulfoxide

**DOI:** 10.1371/journal.ppat.1006428

**Published:** 2017-06-06

**Authors:** Audrey S. Vanhove, Saiyu Hang, Vidhya Vijayakumar, Adam CN Wong, John M. Asara, Paula I. Watnick

**Affiliations:** 1 Division of Infectious Diseases, Boston Children’s Hospital, Harvard Medical School, 300 Longwood Avenue, Boston MA, United States of America; 2 Division of Signal Transduction, Beth Israel Deaconess Medical Center, 3 Blackfan Circle, Boston MA, United States of America; 3 Department of Medicine, Harvard Medical School, Boston MA, United States of America; 4 Department of Microbiology and Immunobiology, Harvard Medical School, 77 Avenue Louis Pasteur, Boston, MA United States of America; McMaster University, CANADA

## Abstract

*Vibrio cholerae* is a diarrheal pathogen that induces accumulation of lipid droplets in enterocytes, leading to lethal infection of the model host *Drosophila melanogaster*. Through untargeted lipidomics, we provide evidence that this process is the product of a host phospholipid degradation cascade that induces lipid droplet coalescence in enterocytes. This infection-induced cascade is inhibited by mutation of the *V*. *cholerae* glycine cleavage system due to intestinal accumulation of methionine sulfoxide (MetO), and both dietary supplementation with MetO and enterocyte knock-down of host methionine sulfoxide reductase A (MsrA) yield increased resistance to infection. MsrA converts both free and protein-associated MetO to methionine. These findings support a model in which dietary MetO competitively inhibits repair of host proteins by MsrA. Bacterial virulence strategies depend on functional host proteins. We propose a novel virulence paradigm in which an intestinal pathogen ensures the repair of host proteins essential for pathogenesis through consumption of dietary MetO.

## Author summary

The virulence program of intestinal pathogens such as *Vibrio cholerae* depends on the continued function of target host proteins. If these proteins are inactivated by methionine oxidation, virulence may also depend on repair of these proteins by host methionine sulfoxide reductases such as MsrA. Dietary methionine sulfoxide competitively inhibits repair of host proteins by MsrA. Here, using the model host *Drosophila melanogaster*, we demonstrate a novel virulence paradigm in which *V*. *cholerae* uptake of dietary methionine sulfoxide frees host MsrA to repair host proteins essential for virulence.

## Introduction

Childhood diarrheal disease is a leading cause of morbidity and mortality, particularly in the developing world, and bacterial pathogens figure prominently in this entity [1]. Traditional virulence factors are pathogen-specific and synthesized expressly for the purpose of colonizing, entering, and manipulating host intestinal epithelial cells. However, well-conserved metabolic pathways of diarrheal pathogens may also contribute to disease by altering the metabolite profile of the intestinal contents, the composition and physiology of the resident intestinal microbiota, and local and systemic host metabolism. This, in turn, may have immediate and long-lasting impacts on host intestinal function and nutrition.

*Vibrio cholerae*, an important pathogen in many regions of the developing world, causes a life-threatening diarrheal disease when ingested in contaminated water or food [2]. Two intensively studied virulence factors of *V*. *cholerae* are the toxin co-regulated pilus and cholera toxin, which is responsible for the severe secretory diarrhea of cholera [2]. To identify and study additional virulence factors in a genetically tractable host, we developed the arthropod *Drosophila melanogaster* as a model for *V*. *cholerae* infection [3]. In our experimental design, *Drosophila* are fed *V*. *cholerae* in LB-broth, and a lethal infection ensues. This infection is independent of the toxin co-regulated pilus and is only partially mitigated by deletion of the genes encoding cholera toxin. *V*. *cholerae* colonizes the fly midgut and rectum, disrupts adherens junctions, suppresses intestinal stem cell division, causes accumulation of large lipid droplets in enterocytes, and suppresses insulin signaling [3–7]. While cholera toxin plays only a small role in pathogenesis in this model, the accumulation of large lipid droplets in *Drosophila* enterocytes is correlated with mortality [3, 6, 7].

We recently reported a *Drosophila*-based screen for *V*. *cholerae* transposon insertion mutants with decreased virulence [7]. This screen identified the virulence factor CrbRS, a two-component system that regulates acetate uptake in a process known as the acetate switch [8]. In response to an unknown signal, CrbRS activates transcription of *acs1*, a gene encoding the enzyme acetyl-CoA synthase, which uses acetate as a substrate. We reported that elimination of intestinal acetate uptake by *V*. *cholerae* prevented lipid droplet formation in enterocytes and interruption of host insulin signaling. In mammals, short chain fatty acids (SCFA) such as acetate, propionate and butyrate, are principally produced by the intestinal microbiota and serve as both signals and nutrients that maintain the intestinal epithelium [9, 10]. Intestinal uptake of SCFA by pathogens suggests a difference in metabolism between pathogenic and commensal bacteria in the intestinal environment and provides one mechanism by which pathogen metabolism may contribute to virulence. Here we report an additional mechanism by which pathogen metabolism may manipulate intestinal function.

We previously identified several *V*. *cholerae* glycine cleavage system mutants that were attenuated for virulence [7]. While exploring the virulence attenuation of *V*. *cholerae* glycine cleavage system mutants, we uncovered an intestinal phospholipid degradation cascade that is responsible for lipid droplet coalescence in enterocytes during infection. Here we report that the host proteins required for this cascade are inactivated by dietary methionine sulfoxide (MetO), disruption of the *V*. *cholerae* glycine cleavage system, and inhibition of host methionine sulfoxide reductase A (MsrA), an enzyme that reduces both free and protein-associated MetO to methionine [11, 12]. In the absence of repair by MsrA, oxidation of exposed methionines can lead to protein inactivation. We propose that free MetO competitively inhibits MsrA-dependent repair of a protein required for *V*. *cholerae*-activated phospholipid degradation. Consumption of dietary methionine sulfoxide by wild-type *V*. *cholerae* relieves this inhibition. This represents a novel virulence paradigm in which an intestinal pathogen, through its metabolism, promotes the repair of host proteins essential for virulence.

## Results

### The *V*. *cholerae* glycine cleavage system is essential for virulence in a *Drosophila* model of cholera

**Fig 1:**
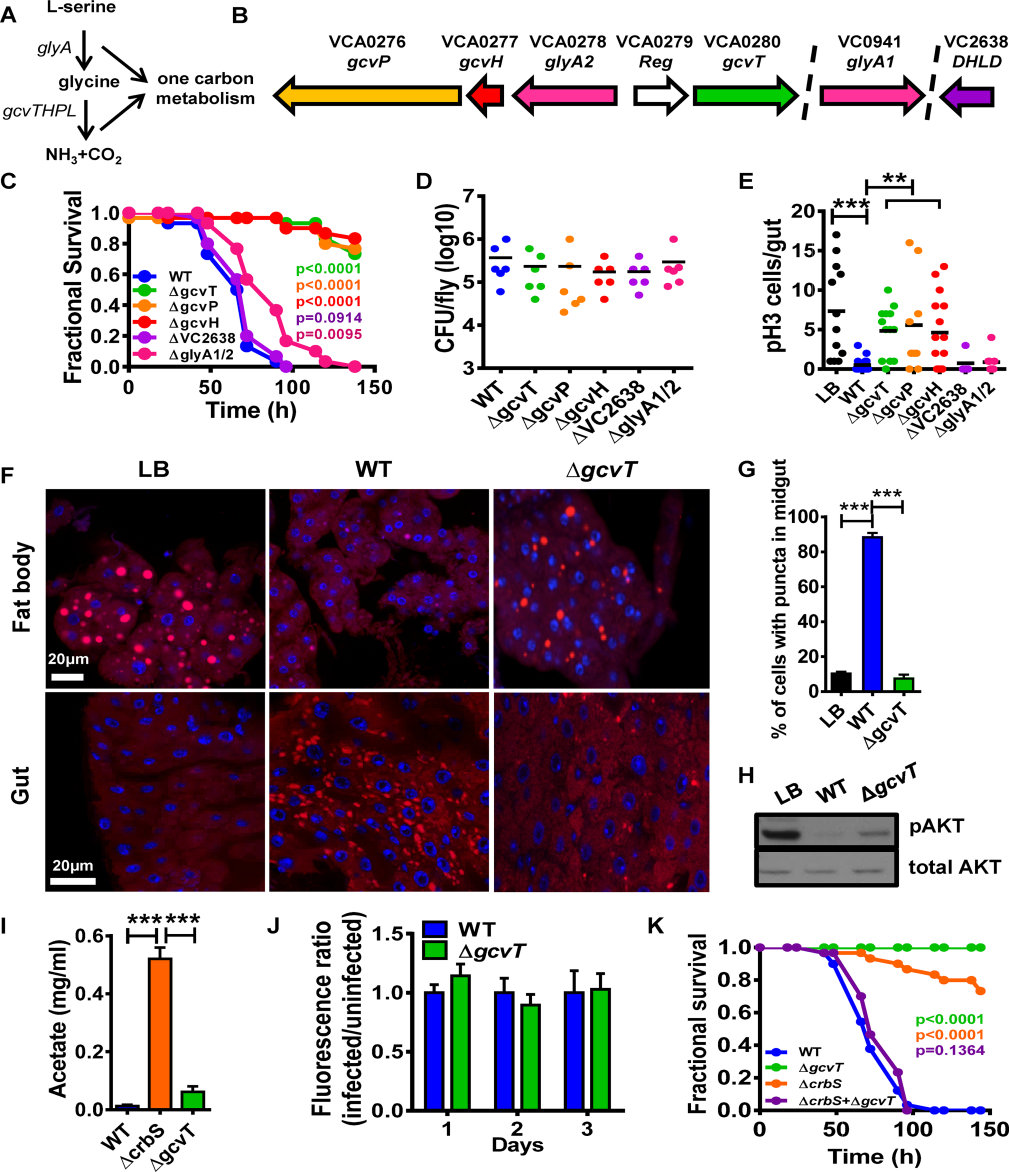
The *V*. *cholerae* glycine cleavage system promotes host metabolic disruption and suppression of intestinal stem cell division by a novel mechanism. (A) Components of the glycine cleavage system. (B) Chromosomal environments of *glyA2*, *gcvH*, *gcvP*, VC2638 and *glyA1*. (C) Survival curves of Oregon R flies fed LB broth inoculated with wild-type *V*. *cholerae* (WT), glycine cleavage system mutants or serine catabolism mutants. (D) *V*. *cholerae* colony-forming units (cfu) per fly after 48h of exposure to LB broth inoculated with the indicated *V*. *cholerae* strains. (E) Enumeration of PH3^+^ cells/fly intestine after 72h of exposure to LB broth alone or inoculated with the indicated *V*. *cholerae* strains. (F) Nile red staining of neutral lipids in the fat body and intestine of flies fed LB broth alone or inoculated with the indicated *V*. *cholerae* strains. (G) Quantification of cells containing lipid droplets in the midgut of flies fed the indicated *V*. *cholerae* strains. (H) Western blot analysis of phosphorylated AKT (pAKT) or total AKT levels in whole flies fed LB broth alone or inoculated with wild-type *V*. *cholerae* (WT) or a Δ*gcvT* mutant. (I) Acetate levels in the spent supernatants of wild-type *V*. *cholerae* (WT) or Δ*crbS* and Δ*gcvT* mutants cultured in LB. (J) Fluorescence ratios of flies fed LB supplemented with fluorescein either alone or inoculated with wild-type *V*. *cholerae* or a Δ*gcvT* mutant and harvested at the indicated time. (K) Survival curves of Oregon R flies fed LB broth inoculated with wild-type *V*. *cholerae* (WT), a Δ*crbS* mutant, a Δ*gcvT* mutant, or a combination of both. For pooled data, the mean and SD are shown. Pairwise statistical significance was calculated using a student’s t-test (*p<0.05, **p<0.01, ***p<0.001). For survival curves, statistical significance was calculated by log-rank analysis.

A genetic screen for *V*. *cholerae* virulence determinants in a *Drosophila* model of infection identified several transposon insertions in genes encoding components of the bacterial glycine cleavage system [7]. The glycine cleavage system, which is highly conserved among bacteria, plants, and animals, is involved in the catabolism of serine and glycine. It consists of the proteins GlyA and GcvH, P, T, and L (Fig 1A) [13]. GlyA is a serine hydroxymethyltransferase that generates glycine from serine along with donation of a methyl group to tetrahydrofolate (THF). GcvH, which is modified with a disulfide bond-containing lipoic acid, activates the glycine decarboxylase GcvP. After release of CO_2_, the glycine-derived reaction intermediate is transferred to a GcvH-associated lipoic acid sulfhydryl group generated by disulfide bond reduction and shuttled to GcvT, an aminomethyltransferase. From here, another methyl group is transferred to THF, and ammonia is released. To re-initiate the cycle, the GcvH-associated lipoic acid disulfide bond must be regenerated by GcvL, a dihydrolipoamide dehydrogenase (DHLD). The methyl groups donated to THF enter the folate cycle, giving rise to key biological compounds such as purines and methionine.

In *V*. *cholerae*, *glyA2*, *gcvH*, *gcvP*, and *gcvT* are found in one chromosomal locus, while VC2638, a putative DHLD and a second *glyA* homolog are found in other regions of the chromosome (Fig 1B).

To explore the role of the glycine cleavage system in virulence, we constructed strains carrying in-frame deletions in *glyA1*, *glyA2*, *gcvH*, *gcvP*, *gcvT*, and VC2638. As shown in Fig 1C, mutations in *gcvT*, *P*, and *H*, but not VC2638 or *glyA1/2* greatly decreased virulence in the fly model. The virulence defect of a Δ*gcvT* mutant could be rescued by an expression plasmid encoding the *gcvT* gene (S1 Fig). This strongly suggests that interference with *V*. *cholerae* glycine but not serine catabolism alters virulence. Furthermore, because the Δ*VC2638* mutant had no virulence defect, we hypothesize that it is not essential for glycine catabolism, possibly due to the presence of another dihydrolipoamide dehydrogenase with redundant function such as that at locus VC2412.

Because our results suggested that the glycine cleavage system was important for virulence, we first tested the possibility that glycine catabolism was required for survival and growth of *V*. *cholerae* within the fly intestine. However, we found that the bacterial burden of strains defective for glycine catabolism was similar to that of the wild-type parental strain (Fig 1D).

### Infection with glycine cleavage system mutants does not suppress host intestinal stem cell division

We previously showed that *V*. *cholerae* infection greatly suppresses intestinal stem cell (ISC) division in the *Drosophila* intestine and that activation of this process prolongs host survival [6]. Therefore, we questioned whether mutation of the glycine cleavage system had an additional effect on ISC division. Histone 3 is phosphorylated during cell division. To assess ISC divisions, we used immunofluorescence to enumerate cells in which histone 3 is phosphorylated in the intestines of uninfected *Drosophila* as well as those infected with wild-type *V*. *cholerae* or a glycine cleavage system mutant. As shown in Fig 1E, *V*. *cholerae* glycine cleavage system mutants with reduced virulence did not suppress ISC division. *V*. *cholerae* Δ*gcvT*, *P*, and *H* mutants had similar infection phenotypes, suggesting a common mechanism of virulence attenuation. Therefore, for simplicity, further investigation of mechanism focused on only one of these, the Δ*gcvT* mutant.

### The intestines of flies infected with a *V*. *cholerae* glycine cleavage system mutant do not accumulate lipid droplets

Infection with wild-type *V*. *cholerae* results in accumulation of lipid droplets in the fly intestine in tandem with depletion of lipid droplets from the *Drosophila* adipose tissue or fat body, and interventions that decrease accumulation of lipid droplets in the intestine prolong host survival [7]. To determine the effect of *Drosophila* infection with a *V*. *cholerae* Δ*gcvT* mutant on lipid droplet distribution, we stained the fat bodies and intestines of flies with the lipophilic dye Nile Red. Similar to what was observed previously, we found that lipid droplets accumulated in the intestines of flies infected with wild-type *V*. *cholerae* and were depleted from the fat body. However, in the absence of *V*. *cholerae gcvT*, none of these derangements were observed (Fig 1F and 1G). We previously observed that depletion of lipid droplets from the *Drosophila* fat body results in diminished signaling through the insulin/insulin-like signaling (IIS) pathway. Activation of the llS pathway results in protein kinase B phosphorylation (p-AKT), which can be detected using a p-AKT-specific antibody. We used Western blot analysis to assess signaling through the llS pathway. As shown in Fig 1H, infection with a *V*. *cholerae* Δ*gcvT* mutant decreased p-AKT to a lesser extent than that with the wild-type strain.

### *V*. *cholerae* glycine cleavage system mutants preserve host viability through a mechanism that is distinct from that of acetate assimilation mutants

In a process known as the acetate switch, *V*. *cholerae* generates acetate through fermentation of available sugars and then consumes acetate when other carbon sources are scarce [7, 8]. We previously showed that *V*. *cholerae* Δ*crbS* mutants, which cannot consume acetate, also have a significant defect in virulence in a *Drosophila* model [7]. To determine if the virulence defect of the glycine cleavage system mutants was due to an inability to consume acetate, we compared acetate concentrations in the spent supernatants of wild-type *V*. *cholerae*, Δ*gcvT* mutant, and Δ*crbS* mutant cultures. As shown in Fig 1I, acetate concentrations in the spent supernatants of Δ*gcvT* mutant cultures were not significantly different from those in wild-type *V*. *cholerae* supernatants. *V*. *cholerae* acetate uptake mutants increase food accumulation in the *Drosophila* intestine suggesting an effect on appetite [7]. Ingestion of the Δ*gcvT* mutant did not have this effect (Fig 1J). These data suggested to us that *V*. *cholerae* glycine cleavage system and acetate uptake pathways are independent mediators of virulence in the fly model.

We reasoned that if mutations in *crbS* and *gcvT* rescued fly survival through the inability to consume distinct nutrients, co-infection with Δ*crbS* and Δ*gcvT* mutants would result in net consumption of all differentially secreted metabolites, leading to a restoration of virulence. In fact, we found that, while each mutant alone had a significant virulence defect, co-infected flies died at the same rate as flies infected with wild-type *V*. *cholerae* (Fig 1K). This is consistent with the hypothesis that Δ*crbS* and Δ*gcvT* mutants rescue virulence through secretion or an inability to utilize distinct small molecules that are normally consumed by *V*. *cholerae*.

### Glycine secretion is not responsible for the decreased virulence of glycine cleavage system mutants

Glycine cleavage system mutants have previously been shown to secrete glycine into the culture medium [14]. As an indication that glycine might be secreted into the fly intestine by the *V*. *cholerae ΔgcvT* mutant, we measured transcription of the *Drosophila gcvH* (*ppl*), *gcvL* (CG7430), *gcvT* (CG6415), and *gcvP* (CG3999) genes in the intestines of LB-fed *Drosophila* and those infected with wild-type *V*. *cholerae* or a *ΔgcvT* mutant (S2A–S2D Fig). We found that infection with wild-type *V*. *cholerae* decreased intestinal transcription of *Drosophila gcvH* and *gcvL*, while infection with the *V*. *cholerae* Δ*gcvT* mutant increased transcription of these genes. We hypothesize that this represents the transcriptional response of the *Drosophila* intestine to consumption and secretion of glycine by wild-type *V*. *cholerae* and the Δ*gcvT* mutant, respectively. We reasoned that if secretion of glycine by the *V*. *cholerae* Δ*gcvT* mutant was responsible for prolonged host survival, diet supplementation with glycine during wild-type *V*. *cholerae* infection should also have this effect. However, we found that glycine ingestion did not alter host survival of infection (S2E Fig). We also administered glycine to flies in phosphate buffered saline (PBS) and measured ISC division. This treatment decreased ISC division (S2F Fig). These results demonstrate that the host intestine senses and responds to glycine secretion by a *V*. *cholerae* Δ*gcvT* mutant, but this response neither increases host resistance to infection nor restores ISC division.

### Metabolomics highlights differences between wild-type *V*. *cholerae* and a glycine cleavage system mutant in culture, in the arthropod intestine, and in the mammalian host

**Fig 2:**
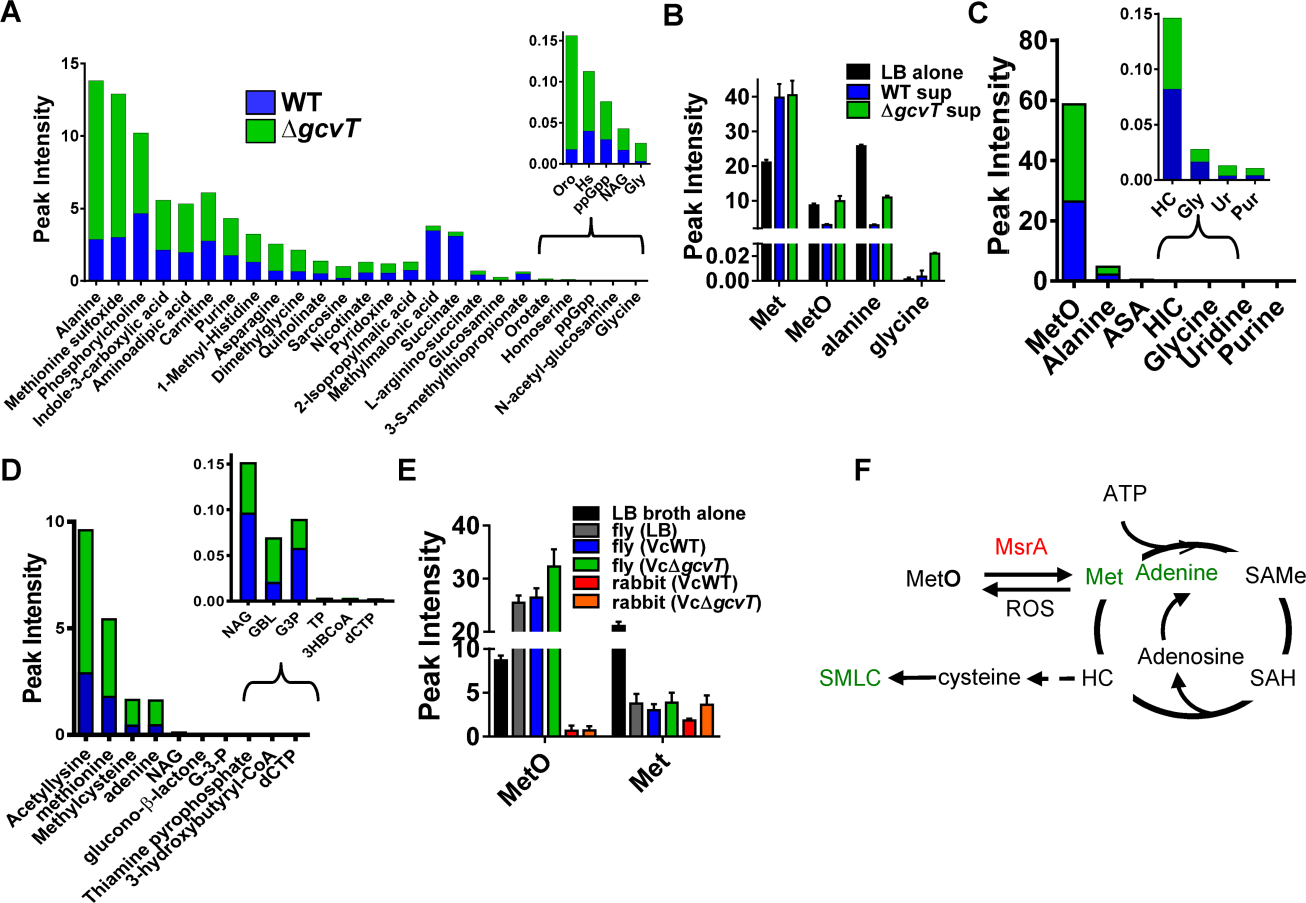
Mutation of the *V*. *cholerae* glycine cleavage system alters the extracellular environment in LB broth, *Drosophila* intestine, and the rabbit intestine. LC-MS/MS based metabolomic analysis of wild-type *V*. *cholerae* (WT) or a Δ*gcvT* mutant in (A and B) LB culture supernatants, (C) infected *Drosophila* intestines, and (D) cecal fluid of infected infant rabbits. Only metabolites that were significantly different under the two conditions are shown (p<0.05). (E) Levels of MetO and methionine (Met) in LB broth alone, in the intestines of flies fed LB broth alone (LB), LB broth inoculated with wild-type *V*. *cholerae* (WT) or LB broth inoculated with a *V*. *cholerae* Δ*gcvT* mutant, and in the cecal fluid of infant rabbits inoculated with wild-type *V*. *cholerae* or a *V*. *cholerae* Δ*gcvT* mutant. (F) Compounds identified in cecal fluid metabolomics are shown in green along with their relationship to the methionine cycle. For pooled data, the mean and SD are shown.

We hypothesized that the metabolic interaction of a glycine cleavage system mutant with its environment might extend beyond secretion of glycine. To test this, we analyzed polar metabolites in sterile LB broth as well as the spent culture supernatants of wild-type *V*. *cholerae* and a Δ*gcvT* mutant by targeted LC-MS/MS (Fig 2A and 2B, and S1 Table) [15]. Alanine and methionine sulfoxide (MetO) were several fold lower in the supernatants of wild-type *V*. *cholerae* as compared with sterile LB broth (Fig 2B). However, levels of these two metabolites in the *V*. *cholerae* Δ*gcvT* mutant supernatant were more like those of sterile LB, suggesting very little consumption of these amino acids by the Δ*gcvT* mutant. As expected, levels of glycine were similar in LB broth and the spent supernatant of wild-type *V*. *cholerae* but higher in that of the Δ*gcvT* mutant (Fig 2B).

To assess the relevance of our findings in culture to the infected *Drosophila* intestine, we performed metabolomics on the intestines of flies fed LB broth alone or inoculated with wild-type *V*. *cholerae* or a Δ*gcvT* mutant (Fig 2C and S2 Table), using a sample preparation protocol that eliminated protein-associated amino acids and metabolites. In general, the differences observed were smaller than those observed in culture (Fig 2A–2C, S3 Fig and S1 and S2 Tables). In an infection model, we reason that nutrients left behind by the pathogen are rapidly consumed by the host. However, similar to our culture results, free alanine and MetO were significantly elevated in the intestines of Δ*gcvT*-infected flies as compared with those of flies infected with wild-type *V*. *cholerae*. Interestingly, in contrast to LB broth, to which both the pathogen and host had access during infection, the level of MetO in the intestines of flies fed LB broth alone or inoculated with *V*. *cholerae* were much higher than that of methionine (Fig 2E). This may reflect a highly oxidizing intestinal environment.

We extended our studies to the mammalian intestine. In the recently resuscitated infant rabbit model of cholera [16], cecal fluid accumulates during infection and is easily harvested. We infected infant rabbits with either wild-type *V*. *cholerae* or a Δ*gcvT* mutant, assessed colonization, and then harvested cecal fluid for metabolomic analysis. Similar to our findings in the fly, no significant difference in colonization or fluid accumulation was observed in the terminal ileum or cecum of rabbits infected with these two strains (S4A–S4C Fig). We collected cecal fluid from infected rabbits, removed cells and other particulates by centrifugation, and then performed metabolomic analysis on the resulting fluid (Fig 2D and S3 Table). While there were very low concentrations of free methionine and MetO in the intestines of infant rabbits as compared with the concentrations of these metabolites in LB broth and the fly intestine (Fig 2E), methionine, methylcysteine, and adenine were more abundant in the cecal fluid of rabbits infected with a *V*. *cholerae* Δ*gcvT* mutant. These metabolites are linked to methionine sulfoxide via methionine sulfoxide reductase and the methionine cycle (Fig 2F), and their presence in the cecal fluid suggests that there is more capacity for MetO reduction and metabolism in the infant rabbit intestine as compared with the adult *Drosophila* intestine. We conclude that mutation of *V*. *cholerae gcvT* alters pathogen methionine metabolism in LB broth, the arthropod intestine, and the mammalian intestine and, therefore, that the *V*. *cholerae* glycine cleavage system has the potential to modulate host intestinal physiology.

### MetO promotes host survival of infection

**Fig 3:**
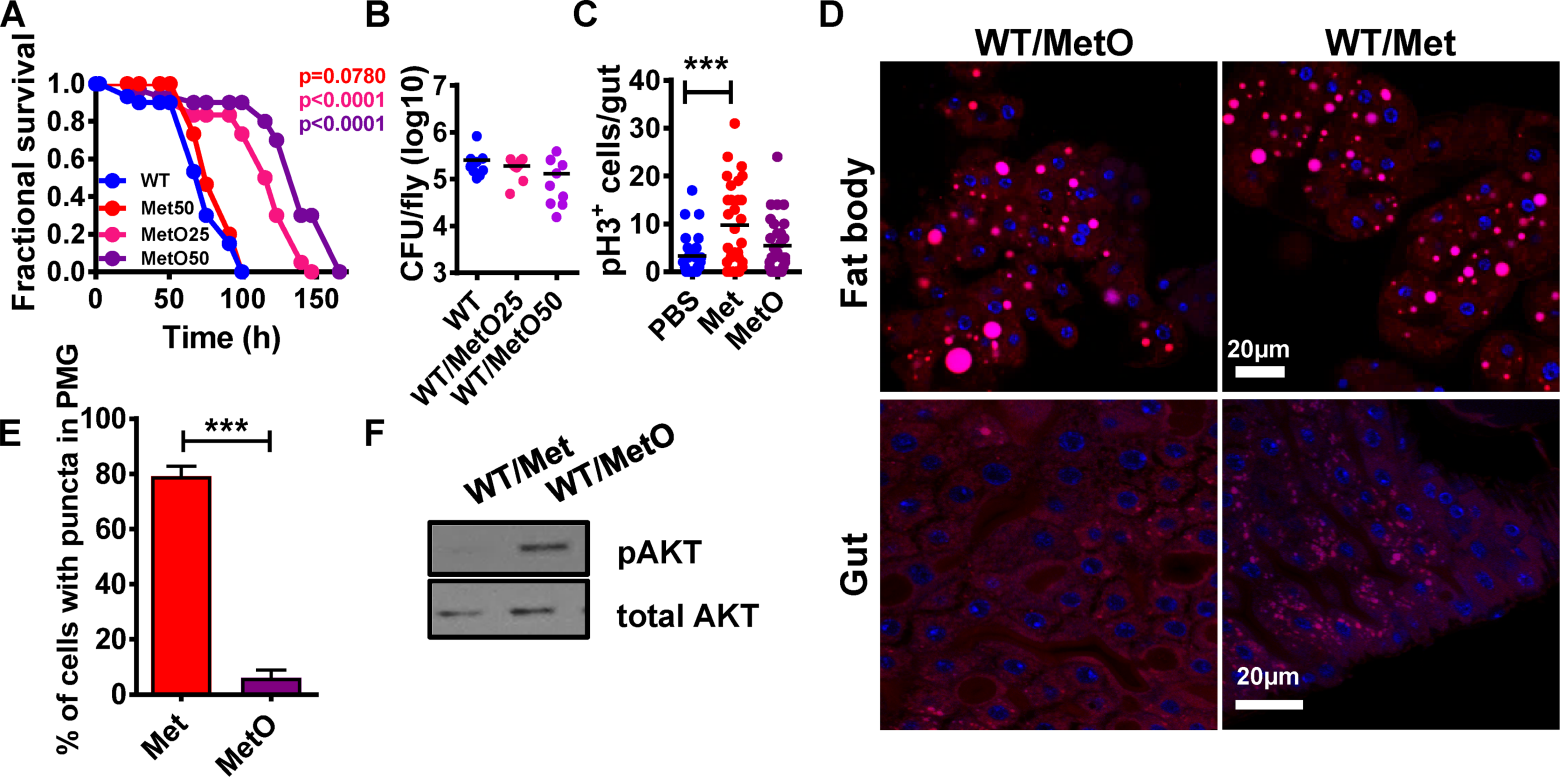
Decreased consumption of methionine sulfoxide (MetO) by the *V*. *cholerae* Δ*gcvT* mutant attenuates virulence by promoting intestinal lipid mobilization and insulin signaling. (A) Survival curves of flies fed LB broth inoculated with wild-type *V*. *cholerae* (WT) supplemented with 50 mM methionine (Met) or 25 mM and 50 mM methionine sulfoxide (MetO). (B) Bacterial burden of flies measured in colony forming units (cfu) after ingestion of LB broth supplemented with 25mM and 50mM methionine sulfoxide (MetO) and inoculated with wild-type *V*. *cholerae* (WT). (C) Enumeration of PH3^+^ cells in the intestines of flies fed PBS or PBS supplemented with 50 mM methionine (Met) and 50 mM methionine sulfoxide (MetO) for 72h. (D) Nile red staining of neutral lipids in the fat body and intestine of flies fed LB broth or LB broth inoculated with wild-type *V*. *cholerae* (WT) supplemented with methionine (Met) or methionine sulfoxide (MetO). (E) Quantification of midgut cells containing lipid droplets. (F) Western blot analysis of phosphorylated AKT (pAKT) or total AKT levels in whole flies fed LB broth alone or inoculated with wild-type *V*. *cholerae* (WT) supplemented with methionine (Met) or methionine sulfoxide (MetO). For pooled data, the mean and SD are shown. Pairwise statistical significance was calculated using a student’s t-test (*p<0.05, **p<0.01, ***p<0.001).

Alanine and MetO were more abundant in the intestines of flies infected with the Δ*gcvT* mutant as compared with wild-type *V*. *cholerae*. We then explored whether diet supplementation with these amino acids might increase host resistance to infection (Figs 3A and S5A). While MetO significantly prolonged fly survival, alanine and a number of other metabolites identified had no effect. Importantly, MetO did not significantly affect *V*. *cholerae* growth either in LB or in the fly and did not impact fly mortality in the absence of infection (Figs 3B, S5B and S5C). These data suggest that a decrease in MetO uptake as a result of mutation of *V*. *cholerae gcvT* leads to an interaction that is more favorable for the host.

Free methionine as well as protein-associated methionine is easily oxidized to MetO [17]. This occurs *in vivo* through exposure to reactive oxygen species. The reverse process, conversion of MetO to methionine, is carried out by methionine sulfoxide reductases that are widely conserved and present in both flies and bacteria [18, 19]. To determine whether the impact of MetO supplementation on infection was direct or attributable to increased methionine availability, we performed an infection in LB supplemented with methionine. We found that methionine supplementation had no effect on bacterial growth or survival of *V*. *cholerae-*infected flies (Figs 3A, S5C and S5D). These data suggest that MetO but not methionine promotes host survival of *V*. *cholerae* infection.

### Methionine but not metO fuels ISC division

Wild-type *V*. *cholerae* but not Δ*gcvT* mutant infection suppresses ISC division in the *Drosophila* gut. We hypothesized that this might be the result of MetO fueling the methionine cycle through conversion to methionine. We tested this by measuring dividing cells in the intestines of flies fed PBS supplemented with either MetO or methionine. Methionine but not MetO increased ISC division (Fig 3C). Therefore, while dietary methionine stimulates intestinal stem cell division, intestinal MetO is not likely to be the cause of increased ISC division in flies infected with a *V*. *cholerae* Δ*gcvT* mutant.

### Methionine sulfoxide reverses intestinal lipid droplet accumulation

Infection with wild-type *V*. *cholerae* but not a *V*. *cholerae* Δ*gcvT* mutant leads to accumulation of lipid droplets in the intestine, depletes lipid droplets in the fat body, and suppresses signaling through the insulin pathway (IIS). We reasoned that if MetO supplementation prolonged fly survival by the same mechanism as mutation of *V*. *cholerae gcvT*, it would also reverse the metabolic phenotype of flies infected with wild-type *V*. *cholerae*. To assess lipid redistribution, we used Nile Red to stain lipid droplets in the fat bodies and intestines of flies infected with wild-type *V*. *cholerae* alone or supplemented with methionine (Met) or MetO. MetO prevented loss of lipid droplets from the fat body and lipid accumulation in the intestine (Fig 3D and 3E). To assess the impact of MetO on signaling through the IIS pathway, we used Western blot analysis to estimate the abundance of p-AKT in wild-type *V*. *cholerae*-infected flies supplemented with this amino acid. As shown in Fig 3F, supplementation with MetO preserved signaling through the IIS pathway. In contrast, both in the presence and absence of infection, supplementation with Methionine increased lipid droplets in the gut without depleting lipids in the fat body or decreasing insulin signaling (Figs 3D–3F and S5E–S5G). This suggests that depletion of lipids from the fat body deactivates insulin signaling during *V*. *cholerae* infection.

Lipid droplets are also present in Human Embryonic Kidney 293 cells (HEK293 cells). To determine whether the effect of MetO was specific to fly enterocytes, we incubated these human cells with MetO and the hydrophobic fluorescent dye boron-dipyrromethene (BODIPY) and enumerated lipid droplets. As shown in S5H and S5I Fig, fewer lipid droplets were also observed in human cells incubated with MetO.

Taken together, these data suggest that, similar to infection with a *V*. *cholerae* Δ*gcvT* mutant, MetO but not methionine supplementation promotes host survival by limiting intestinal lipid droplet accumulation.

### *V*. *cholerae* infection activates host intestinal phospholipid degradation

**Fig 4:**
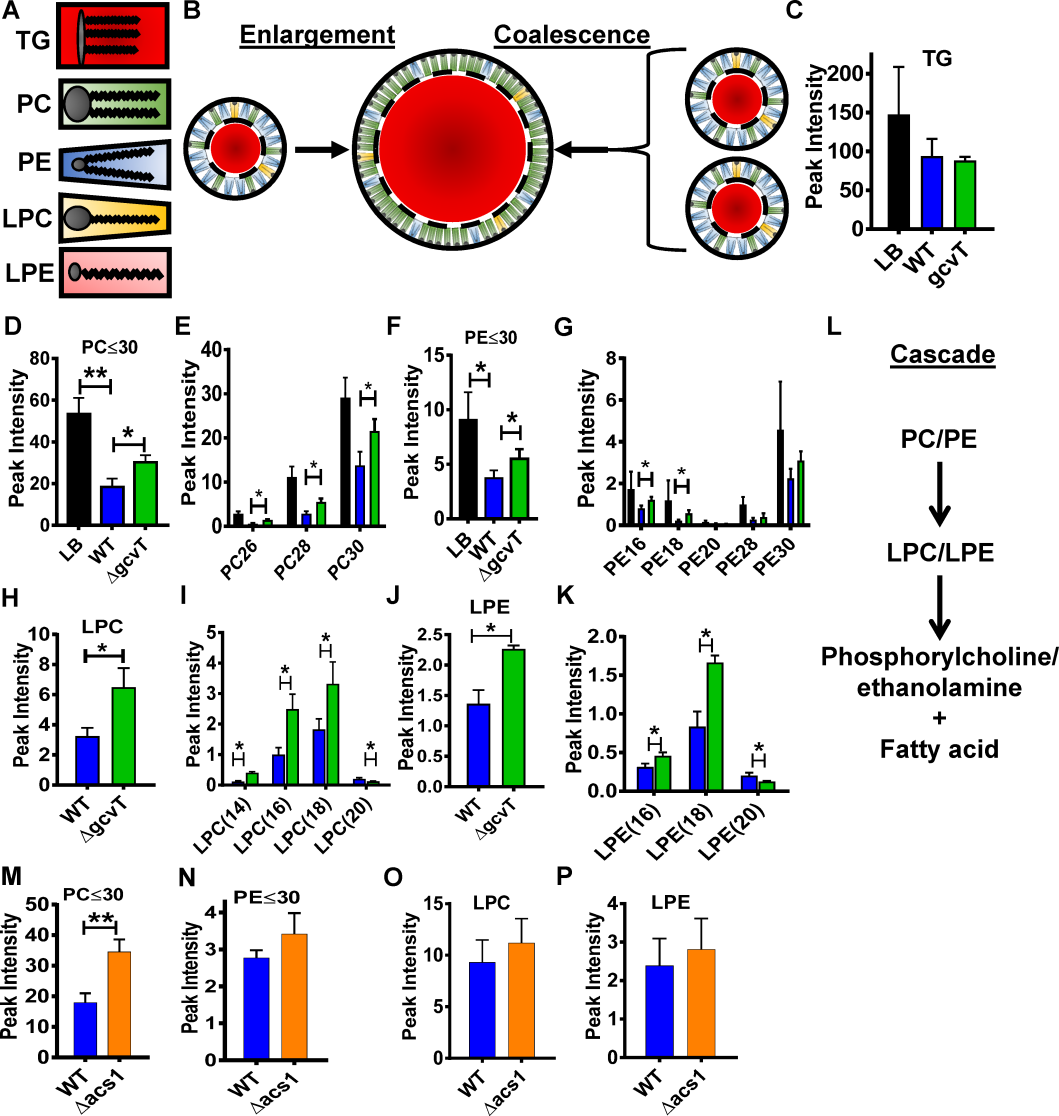
Infection decreases short chain phospholipids in the intestines of *V*. *cholerae*-infected flies. **Lysophospholipids are more abundant in the intestines of Δ*gcvT* mutant infected flies.** (A) Schematic representation of the lipid species discussed in the text. Grey circles represent the polar headgroups, lines represent the fatty acid chains, and surrounding shaded shapes represent the relative space filled by the head group and fatty acid chains of each lipid species. The larger the area of the head group relative to the area filled by fatty acid chains, the greater the propensity to form a highly-curved structure such as a small lipid droplet. (B) Two mechanisms by which small lipid droplets may form a larger lipid droplet are illustrated. Enlargement, shown on the left, results when a large influx of triglycerides must be accommodated. Coalescence results when the supply of phospholipids is inadequate to coat the triglyceride core, and smaller lipid droplets join to minimize exposed surface area. (C-K) LC-MS/MS-based lipidomic analysis of the intestines of flies fed LB alone or inoculated with wild-type *V*. *cholerae* (WT) or a Δ*gcvT* mutant. (C) Triglycerides (TG), (D) Total phosphatidylcholine species with a total of 30 carbons or less in the two fatty acid chains (PC≤30), (E) Individual phosphatidylcholine species with the total number of fatty acid carbons indicated below. (F) Total phosphatidylethanolamine species with a total of 30 carbons or less in the two fatty acid chains (PE≤30) (G) Individual phosphatidylethanolamine species with the total number of fatty acid carbons indicated below. (H) Total lysophosphatidylcholine species. (I) Individual lysophosphatidylcholine species with the total number of fatty acid carbons indicated below. (J) Total lysophosphatidylethanolamine species. (K) Individual lysophosphatidylethanolamine species with the total number of fatty acid carbons indicated below. (L) Putative phospholipase cascade. (M-P) Lipidomic analysis of the intestines of flies infected with wild-type *V*. *cholerae* (WT) or a *Δacs1* mutant. (M) Total phosphatidylcholine species with fatty acid carbons less than or equal to 30 (PC≤30). (N) Total phosphatidylethanolamine species with fatty acid carbons less than or equal to 30 (PE≤30). (O) Total lysophosphatidylcholine species. (P) Total lysophosphatidylethanolamine species. For pooled data, the mean and SD are shown. Pairwise statistical significance was calculated using a student’s t-test (*p<0.05, **p<0.01, ***p<0.001).

Lipid droplets consist of an inner triglyceride core and an outer monolayer of polar phospholipids that forms the interface between the hydrophilic cell cytoplasm and the hydrophobic triglyceride core [20, 21]. The radius of curvature accommodated by these phospholipids depends on the size of the polar headgroup relative to the hydrophobic fatty acid chains. Phospholipids with larger head groups and shorter fatty acid chains or just one fatty acid chain such as a lysophospholipid form smaller lipid droplets (Fig 4A). We reasoned that large lipid droplets in the intestines of *Drosophila* infected with *V*. *cholerae* could result either from an increase in triglycerides within enterocytes leading to lipid droplet enlargement or from a decreased supply of short chain phospholipids leading to lipid droplet coalescence (Fig 4B). To distinguish between these two possibilities, we performed untargeted LC-MS/MS lipidomic analysis on the intestines of flies fed LB broth alone or inoculated with wild-type *V*. *cholerae* to quantify a vast array of lipid classes and fatty acids (S6 Fig and S4 Table). While triglyceride levels did not change with infection (Fig 4C), we noted a large decrease in short chain phosphatidylcholine (PC) and phosphatidylethanolamine (PE) species with total fatty acid carbons totaling less than or equal to 30 (Fig 4D–4G). This suggests that coalescence of lipid droplets in the gut is precipitated by catabolism of short chain phospholipids.

### The intestinal phospholipid profile of flies infected with a *V*. *cholerae* Δ*gcvT* mutant suggests a lysophospholipid intermediate

To explore the increased survival of *Drosophila* infected with a *V*. *cholerae* Δ*gcvT* mutant, we then applied untargeted lipidomics to the intestines of these flies (S7 Fig and S5 Table) [22]. Interestingly, we again observed no difference in triglyceride levels (Fig 4C). However, a significantly larger amount of short chain PE and PC was detected in flies infected with the Δ*gcvT* mutant as compared with those infected with wild-type *V*. *cholerae* (Fig 4D–4G). Interestingly, we also observed an accumulation of lysophosphatidylcholine (LPC) and lysophosphatidylethanolamine (LPE) (Fig 4H–4K), which are phospholipids from which one fatty acid chain has been enzymatically removed (Fig 4A). We hypothesize that *V*. *cholerae* infection induces an intestinal phospholipid degradation cascade and that this cascade is partially blocked by infection with a *V*. *cholerae* Δ*gcvT* mutant resulting in accumulation of lysophospholipid intermediates (Fig 4L). To demonstrate that this intestinal phospholipid profile was unique to *V*. *cholerae* infection attenuated by mutation of *gcvT*, we additionally explored the intestinal phospholipid profile of flies infected with the attenuated *V*. *cholerae* Δ*acs1* mutant (S8 Fig and S4 Table). In this case, we observed that, similar to a *V*. *cholerae* Δ*gcvT* infection, the level of short chain PC’s was increased as compared with wild-type *V*. *cholerae* (Fig 4M). However, in contrast to the Δ*gcvT* infection, there was no difference in PE, LPC, or LPE (Fig 4N–4P). This demonstrates that resistance to infection is correlated with inhibition of short chain phospholipid degradation in the intestine and also that *V*. *cholerae gcvT* and *acs1* mutations alter the host intestinal phospholipid profile in unique ways.

### Host methionine sulfoxide reductase A is required for lethal *V*. *cholerae* infection

**Fig 5:**
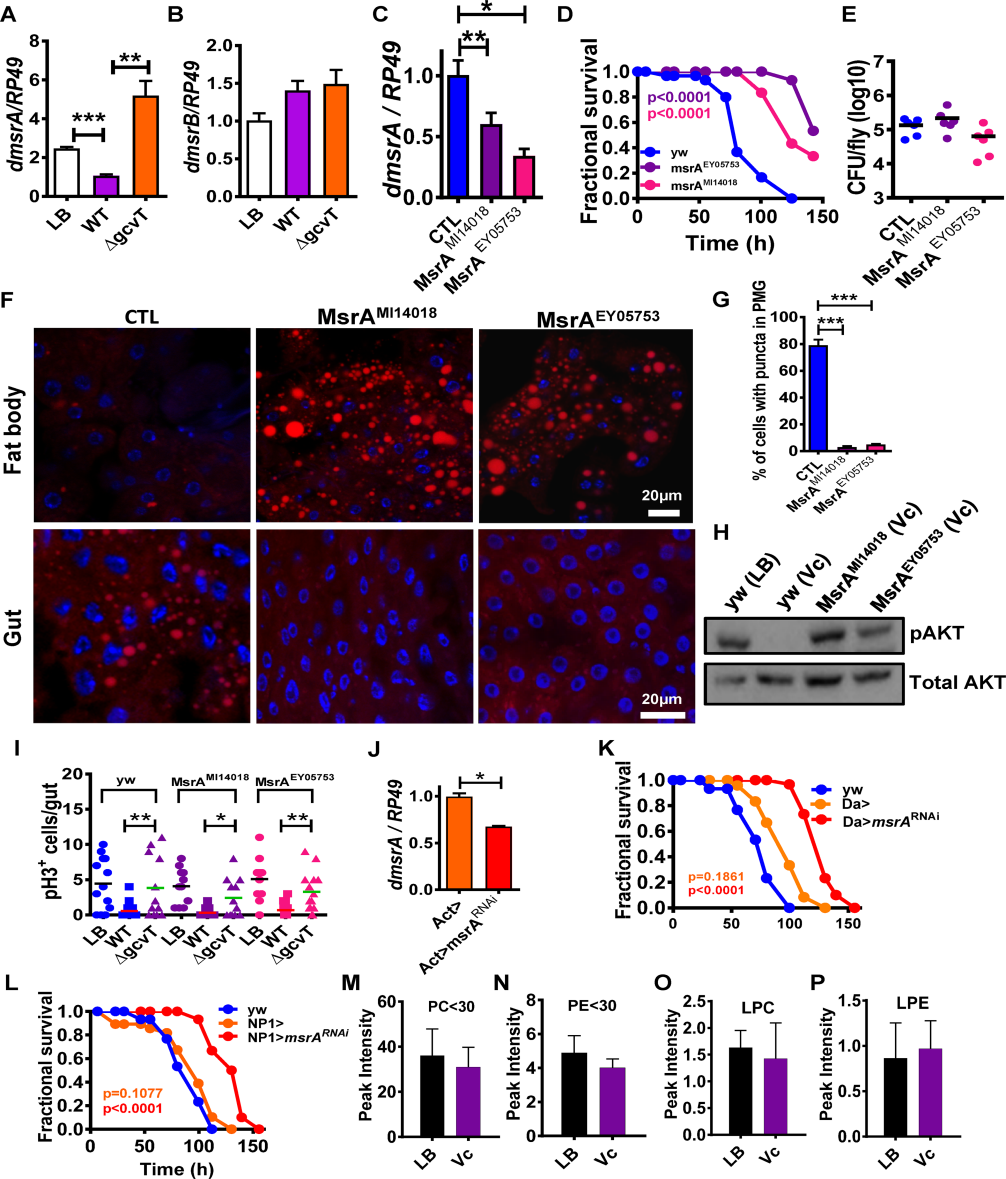
Inactivation of host methionine sulfoxide reductase in the setting of wild-type *V*. *cholerae* infection phenocopies infection of control flies with a *V*. *cholerae* Δ*gcvT* mutant. Transcription levels of (A) d*msrA* and (B) *dmsrB* in the intestines of flies infected with wild-type *V*. *cholerae* (WT) or a Δ*gcvT* mutant. (C) Transcriptional levels of *msrA* in control flies (yw) as well as fly lines carrying the two mutant alleles studied here, *msrA*^*MI14018*^ and *msrA* ^*EY05753*^. (D) Fractional survival of control (yw) or d*msrA* mutant flies infected with wild-type *V*. *cholerae*. (E) Bacterial burden of control (yw) flies and *dmsrA* mutants after infection with *V*. *cholerae*. (F) Nile red staining of neutral lipids in the fat body and intestine of wild-type (yw) or d*msrA* fly mutant flies fed wild-type *V*. *cholerae*. (G) Quantification of midgut cells containing lipid droplets. (H) Western blot analysis of phosphorylated AKT(pAKT) or total AKT levels in whole control (yw) or d*msrA* mutant flies fed LB broth alone (LB) or inoculated with wild-type *V*. *cholerae* (Vc). (I) Enumeration of PH3^+^ cells in the intestines of control (yw) or d*msrA* mutant flies fed LB broth alone or inoculated with wild-type *V*. *cholerae* (WT) or a Δ*gcvT* mutant at 72h. (J) Levels of *msrA* transcription in control (Act-Gal4>) and Act-Gal4>*msrA*^V48990^-RNAi flies. (K) Fractional survival of control (yw), Da-Gal4>, or Da-Gal4>*msrA* ^V48990^-RNAi flies infected with wild-type *V*. *cholerae*. (L) Fractional survival of control (yw), NP1>, or NP1>*msrA* ^V48990^-RNAi flies fed with wild-type *V*. *cholerae*. (M-P) Lipidomics analysis of the intestines of *msrA* ^*EY05753*^ flies fed LB alone (LB) or inoculated with wild-type *V*. *cholerae* (Vc). (M) Total phosphatidylcholine species with fatty acid carbons less than or equal to 30 (PC≤30). (N) Total phosphatidylethanolamine species with fatty acid carbons less than or equal to 30 (PE≤30). (O) Total lysophosphatidylcholine species. (P) Total lysophosphatidylethanolamine species. For pooled data, the mean and SD are shown. Pairwise statistical significance was calculated using a student’s t-test (*p<0.05, **p<0.01, ***p<0.001). Log-rank analysis for survival curves (*p<0.05, **p<0.01, ***p<0.001).

Our data suggested that MetO rather than methionine was critical for resistance to infection. Both free and protein-associated MetO is abundant in oxidizing environments such as the fly intestine. For this reason, animals and bacteria alike possess intracellular methionine sulfoxide reductases that reduce MetO to methionine [23]. The fly genome encodes two stereospecific methionine sulfoxide reductases, MsrA and B. MsrA (CG7266), originally termed Eip71CD in the fly due to its regulation by ecdysone [24], reduces methionine-S-sulfoxide, while SelR or MsrB (CG6584) reduces methionine-R-sulfoxide [25]. While both proteins can reduce free MetO, studies in yeast and mammals show that MsrA reduces free MetO much more efficiently than MsrB [26]. We hypothesized that higher levels of MetO in the intestines of Δ*gcvT* mutant-infected flies might alter *Drosophila msrA* and/or *msrB* transcription. In fact, we found that *Drosophila msrA* transcription was increased 5-fold in a Δ*gcvT* mutant infection (Fig 5A). In contrast, transcription of *Drosophila msrB* was unchanged (Fig 5B). This suggested to us that the interaction of *Drosophila* with the *V*. *cholerae* Δ*gcvT* mutant might hinge on host MsrA.

We first characterized two *Drosophila* transposon insertion mutants. The Mi{MIC}Eip71CD^MI14018^ transposon is inserted in a C-terminal region of MsrA that is predicted to be non-coding [27]. The P{EPgy2}Eip71CD^EY05753^ transposon is inserted near the start of the second MsrA intron [28]. We tested the susceptibility of the *msrA*^MI14018^ and *msrA*^EY05753^ mutant fly lines to infection with wild-type *V*. *cholerae* after confirming decreased transcription of *msrA* in these lines (Fig 5C). As shown in Fig 5D, both mutants were highly resistant to infection as compared with controls in spite of similar bacterial burdens (Fig 5E). Infected *msrA* mutant flies showed normal lipid droplet accumulation in the fat body and enterocytes and active insulin signaling as compared with control flies (Fig 5F–5H). ISC division was equally suppressed in control and *msrA* mutant flies (Fig 5I). As an additional test, we obtained a fly line carrying RNAi targeting *msrA* and confirmed knockdown of *msrA* by a ubiquitous driver (Fig 5J). We then tested the effect of *msrA* knockdown on fly survival when the RNAi was driven ubiquitously or specifically to enterocytes (Fig 5K and 5L). In each case, the flies were resistant to infection as compared with driver-only controls. Taken together, these results show that MetO supplementation or *msrA* inactivation in enterocytes limits intestinal lipid droplet size leading to survival of infection.

We hypothesized that lipidomic analysis of the intestines of *msrA* mutant flies fed LB broth alone or inoculated with wild-type *V*. *cholerae* might shed light on the mechanism by which mutation of host MsrA prevents lipid droplet coalescence in the intestine. In an MsrA mutant fly, levels of short chain PC, PE, LPC and LPE did not decrease in response to *V*. *cholerae* infection (Fig 5M–5P and S6 Table).

**Fig 6:**
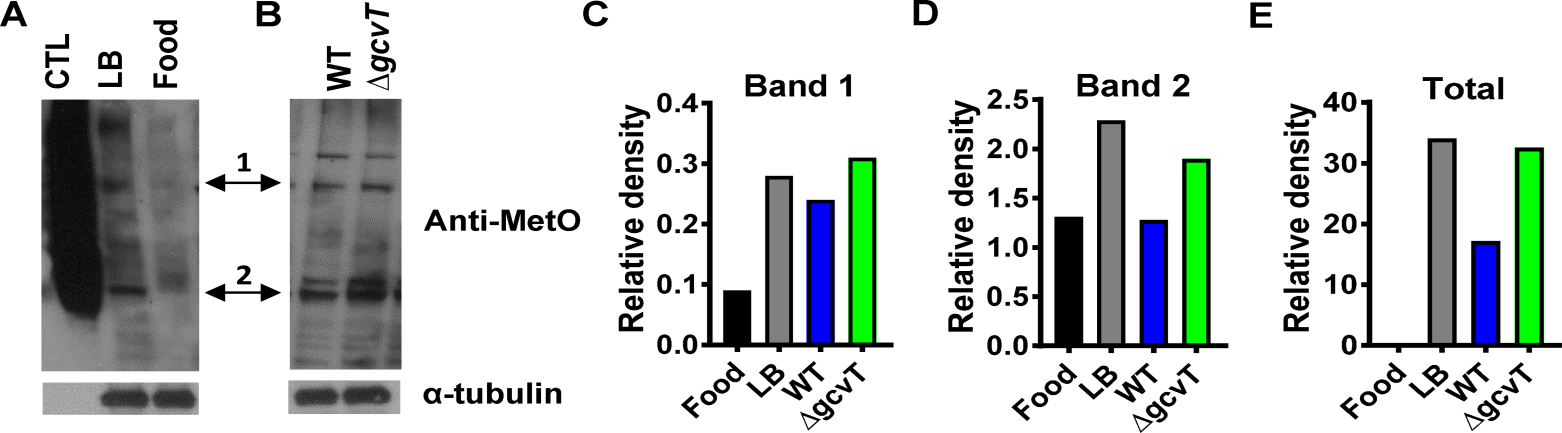
As compared with infection with wild-type *V*. *cholerae*, *Drosophila* infection with a *V*. *cholerae* Δ*gcvT* mutant results in an increase in oxidation of protein-associated methionine within the intestine. Western blot analysis of proteins in the intestines of *Drosophila* fed (A) conventional LB broth (LB) and fly food (Food) or (B) a wild-type *V*. *cholerae* (WT) and a Δ*gcvT* mutant. An antibody recognizing oxidized methionine (anti-MetO) was used. BSA-MetO protein was used as a positive control (CTL), and tubulin was used as a loading control (Tub). (C-E) Densitometry analysis of indicated bands and entire lanes (Total) for Western blots shown in (A) and (B). Relative density represents the density measurement for the individual band or lane normalized to the density measurement of the relevant tubulin loading control. Experimental replicates were performed with similar trends noted.

We hypothesized that, during *V*. *cholerae* infection, proteins essential for the phospholipid degradation cascade defined here depend on repair by host MsrA. However, infection with a Δ*gcvT* mutant results in competitive inhibition of MsrA and increased protein oxidation. To assess the extent of protein oxidation in the *Drosophila* intestine, we harvested intestines and performed Western blot analysis using an antibody reported to recognize MetO [29]. LB broth has been shown to activate dual oxidase in *Drosophila* enterocytes, resulting in a highly oxidizing environment [30]. Therefore, to test whether this antibody could detect protein-associated MetO in the *Drosophila* intestine, we first performed Western blot analysis on the intestines of flies fed fly food or LB broth. As shown in Fig 6A, many more bands were observed in the samples prepared from flies fed LB broth, consistent with a higher amount of protein-associated MetO. Furthermore, densitometry quantification both of specific bands and total staining was increased for samples prepared from flies fed LB broth as compared with those fed fly food (Fig 6C–6E). We then performed the same experiments with flies fed wild-type *V*. *cholerae* or a Δ*gcvT* mutant. Although the difference was subtler in this case, samples prepared from the intestines of flies infected with a Δ*gcvT* mutant produced darker bands on Western blot analysis (Fig 6B). Densitometry analysis again supported our subjective observations (Fig 6C–6E). These results are consistent with our hypothesis that infection with a *V*. *cholerae* Δ*gcvT* mutant leads to decreased repair of protein-associated MetO in the fly intestine.

## Discussion

**Fig 7:**
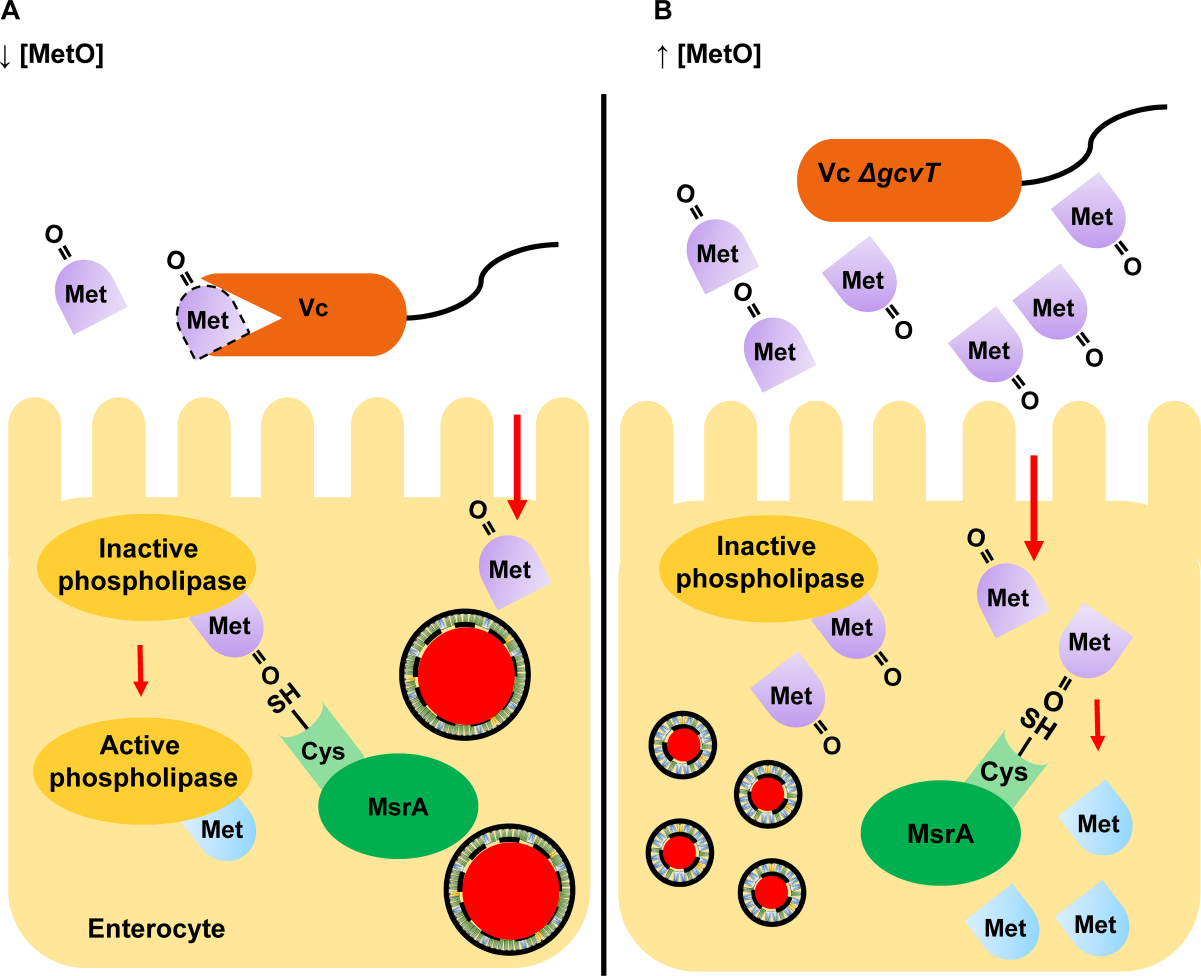
*Vibrio cholerae* augments virulence by modulation of host intestinal methionine sulfoxide reductase. (A) Wild-type *V*. *cholerae* (WT) catabolizes dietary methionine sulfoxide (MetO) in the intestinal lumen, leaving host enterocyte methionine sulfoxide reductase (MsrA) free to repair proteins that have been inactivated by methionine oxidation. (B) In a *V*. *cholerae ΔgcvT* mutant infection, dietary MetO is not consumed by *V*. *cholerae* but rather taken up by host enterocytes. Dietary MetO competitively inhibits reduction of protein-associated MetO by MsrA within enterocytes.

Here we present evidence that *V*. *cholerae* ingestion by the model host *Drosophila melanogaster* activates a phospholipid degradation cascade in enterocytes that results in lipid droplet coalescence, depletion of lipids from adipose tissue, and host death. We show that the activity of this cascade is ensured by *V*. *cholerae* consumption of dietary MetO and is inhibited by knockdown of methionine sulfoxide reductase MsrA within host enterocytes. MsrA is an intracellular protein that reduces dietary and protein-associated MetO to methionine [31]. Protein function can be activated or inactivated by methionine oxidation, and a large body of evidence suggests that reversible oxidation of protein-associated methionine is a mechanism by which cells adjust their physiology in response to reactive oxygen species [32]. Pathogens that co-opt host proteins either through delivery of toxins or type lll secretion system effectors to host cells depend on the continued function of their protein targets for pathogenesis [33, 34]. Based on the findings reported here, we propose a novel mechanism by which a pathogen ensures the continued functioning of host proteins required for virulence during intestinal infection (Fig 7). A large proportion of dietary methionine is consumed in the form of MetO and must be reduced to methionine by MsrA prior to utilization [35]. Thus, dietary MetO competes with protein-associated MetO for reduction by MsrA. During infection, *V*. *cholerae* consumes MetO in the host intestinal lumen. Because very little MetO reaches enterocytes, MsrA is free to reduce protein-associated MetO (Fig 7A). This ensures the continued function of host proteins required for phospholipid degradation and promotes host death. When MsrA expression in enterocytes is decreased by RNAi or the host is infected with a bacterium unable to consume dietary MetO, such as a *V*. *cholerae* Δ*gcvT* mutant, less MsrA is available for repair of host proteins, the phospholipid cascade is blocked, and the host survives (Fig 7B). Therefore, consumption of dietary MetO by *V*. *cholerae* promotes the function of host proteins essential for its virulence.

Our experiments demonstrate that the *V*. *cholerae* glycine cleavage system alters the metabolic profile of the intestinal lumen of flies and rabbits during infection. While methionine and its metabolites were increased in the cecal fluid of rabbits infected with the *V*. *cholerae* Δ*gcvT* mutant, only small amounts of methionine and MetO were present in the cecal fluid of infant rabbits regardless of the infecting *V*. *cholerae* strain. While there are no studies of the composition of rabbit or murine breast milk, methionine is one of the least abundant amino acids in human breast milk [36], and the finding that bacterial methionine synthesis is essential for *V*. *cholerae* colonization of the neonatal mouse intestine suggests that this is also the case in the mouse [37]. While only neonatal mammalian models of cholera are available, the defined diet of neonatal animals underscores a limitation in their use to explore the role of dietary manipulation in protection of young children and adults against cholera.

Our findings may be relevant to human disease. The mechanism we describe for lipid droplet coalescence is also believed to underlie some types of non-alcoholic fatty liver disease (NAFLD) [38]. Furthermore, NAFLD is associated with small intestinal bacterial overgrowth and inflammatory bowel disease, both of which could affect phospholipid genesis, catabolism, and supply in enterocytes and hepatocytes [39–42]. While intestinal or hepatic lipid accumulation has not been explored in cholera, host MsrA and its interaction with luminal MetO may play a role in the osmotic diarrhea of this disease. Calmodulin is a well-established facilitator of secretory diarrhea, and calmodulin antagonists have been developed as anti-diarrheal treatments [43, 44]. This is true, in particular, for cholera, whose secretory diarrhea depends on the CFTR chloride and SK potassium channels, both of which are calmodulin-dependent [45, 46]. Calmodulin is a Ca^2+^*-*binding regulatory molecule, which becomes unresponsive to Ca^2+^ activation upon oxidation of methionines 144 and 145 [47, 48]. Function is restored when these methionines are reduced by MsrA [49, 50]. Our findings suggest a rationale for the investigation of dietary MetO as an inhibitor of cholera toxin-induced diarrhea.

Diarrheal disease is responsible for 1.7 billion childhood infections per year worldwide, which may cause death or lead to life-altering sequelae such as undernutrition, growth faltering, cognitive impairment, poor response to childhood vaccines, and increased risk of death from other causes [1]. Here we describe a novel virulence mechanism by which intestinal microbes may disrupt enterocyte lipid metabolism and diarrheal pathogens may prolong secretory diarrhea. This mechanism points to MsrA as a new target for metabolic and anti-diarrheal treatments and also suggests inexpensive dietary interventions such as MetO supplementation to mitigate disease.

## Materials and methods

### *Drosophila*, bacteria, and human cell culture conditions

*Drosophila* were maintained on Bloomington formulation medium at 25°C. Fly strains used are listed in S7 Table. Where not otherwise noted, L-amino acids and metabolites (Sigma) were used at a concentration of 50 mM. *Vibrio cholerae* strains were cultured in Luria-Bertani (LB) broth or on LB agar supplemented with streptomycin (100 μg/ml) at 27°C. *E*. *coli* strains were grown in LB broth supplemented with ampicillin (100 μg/ml) when necessary at 37°C. Bacterial strains used are listed in S8 Table. Human Embryonic Kidney 293 cells (HEK293 cells, Thermo Fisher Scientific) were cultured in Dulbecco's Modified Eagle Medium (DMEM, Corning) supplemented with 10% fetal bovine serum (Cyclone). For experiments, cells were seeded into 96-well plates at a density of 50,000 cells/well. Where indicated, cells were supplemented with L-methionine sulfoxide (Sigma) (100 mM).

### Bacterial mutagenesis

Mutagenesis was performed by double homologous recombination as previously described [51]. Plasmids and strains used are listed in S8 Table.

### *Drosophila* infections

Infections were performed at 25°C, as previously described [3]. Groups of thirty 5–10 day old female flies were infected with the indicated strains of *V*. *cholerae*. For each condition, flies were divided into three groups of ten and placed in vials containing a cellulose plug infiltrated with 3 mls of LB broth inoculated with a 10-fold dilution of an overnight culture of *V*. *cholerae* and chemicals as noted. For rescue experiments using pBAD expression vectors, LB broth was also supplemented with 100 μg/ml ampicillin and 0.2% L-arabinose. Mortality was enumerated at least once each day. A non-parametric Kaplan–Meier test was used to estimate log-rank values.

### Experimental infection of infant rabbits

1 to 2 day old New Zealand white rabbit kits (Charles river Research Models & Services) were given two doses of oral cimetidine (50mg/kg) 24 and 3 hours prior to administration of the noted *V*. *cholerae* strain. Bacteria were prepared from an overnight culture grown at 30°C. 200μl of a 10^10^ cfu/ml suspension of *V*. *cholerae* in sodium bicarbonate buffer (2.5 g in 100ml; pH 9) was administered to rabbit kits by gavage using 3.5 Fr red rubber catheter. The kits were inspected individually for signs of trauma or aspiration immediately following gavage and then observed periodically for 18 hours post inoculation to monitor dehydration, diarrhea and progressing symptoms. At the first sign of dehydration, kits were sacrificed. Cecal fluid was processed as described below, and intestinal tissues were collected, homogenized and plated to quantify colonization.

### Acetate measurements

The acetate concentration in spent supernatants was measured using an Acetic Acid Assay Kit (Megazyme International Ireland) according to the manufacturer’s instructions. Bacteria were cultured overnight in LB broth, diluted into fresh LB broth to yield a starting OD_600_ of 0.04, and then incubated at 37°C with shaking overnight. 100 μl of this culture was centrifuged to remove bacteria, and the resulting supernatant was diluted in a 1:5 ratio with water and used in the assay. Sodium acetate was used to generate a standard curve.

### Measurements of bacterial load in the fly

For each infection, sixty 7 day old flies were divided equally into six separate vials containing a cellulose plug infiltrated with 3 mls of LB broth into which 300 μl of an overnight culture of the indicated strain had been added. After two days, flies were collected, rinsed in 70% ethanol to remove or lyse bacteria attached to the fly exterior, and homogenized in PBS. Serial dilutions of this suspension were plated on LB agar supplemented with streptomycin (100 μg/ml). After overnight incubation at 27°C, the resulting colonies were enumerated.

### Quantification of food intake

This was carried out as previously described [7]. Briefly, thirty flies divided into three vials per conditions were given access to LB broth alone or inoculated with the indicated strain of *V*. *cholerae* and supplemented with 1% fluorescein (Sigma) for the length of time noted. Ten flies were then washed, homogenized, and centrifuged. The fluorescence intensity of the resulting supernatants was recorded using a microplate spectrophotometer with fluorescence capability (Infinite 200, Tecan). Measurements were normalized to the fluorescence levels of flies fed LB alone and reported as a fluorescence ratio.

### Quantification of gene-specific mRNA levels in *Drosophila*

Thirty to forty-five female flies divided equally into three vials considered experimental replicates, treated as indicated, and harvested for mRNA quantification. For validation of RNAi constructs, whole flies were used. For intestine-specific transcription, intestines were dissected and removed 42 hours after exposure to *V*. *cholerae*. RNA was extracted using a High Pure RNA isolation kit (Roche Life Science) and treated with TURBO DNase treatment (Ambion). Quantification of total RNA was done with a NanoDrop 1000 spectrophotometer (Thermo Fisher Scientific), and quality was monitored by agarose gel electrophoresis. 500 ng of the resulting RNA was used for cDNA synthesis using a Quantitech Reverse transcription kit (Qiagen). Real time q-PCR was performed on the StepOnePlus real-time PCR system (Applied Biosystems) using iTaq Universal SYBR Green supermix (Bio-Rad). Relative expression was calculated using the 2-ΔΔCq method. RP49 (CG7939) gene transcription levels were used for normalization. Primers used are listed in S9 Table.

### Western blot analysis

*Detection of p-AKT*: After three days of exposure to *V*. *cholerae*, 10 flies were homogenized in PBS (100 μl) and heated at 95°C for 15 min. Proteins in the resulting lysates were separated on a 12% SDS-PAGE gel (Biorad) and transferred to a PVDF membrane (Biorad) for hybridization. Primary antibodies were used in the following dilutions: Rabbit anti-Akt: 1:1,000 and Rabbit anti-phospho-Drosophila-Akt (Ser505): 1:1,000 (Cell Signaling Technology). HRP-conjugated anti-rabbit IgG was used in a 1:5,000 dilution as a secondary antibody (Cell Signaling Technology). *Detection of MetO***:** After three days of exposure to fly food, LB, wild-type *V*. *cholerae*, or a Δ*gcvT* mutant, the intestines of 30 flies per condition were isolated, homogenized in PBS (100 μl), and heated at 95°C for 15 min. Proteins in the resulting lysates were separated on a 12% SDS-PAGE gel (Biorad) and transferred on to a PVDF membrane (Biorad) for hybridization. A methionine sulfoxide polyclonal antibody (Cayman) was used in a dilution of 1:200 as a primary antibody and an HRP-conjugated anti-rabbit IgG antibody (Cell Signaling Technology) was used in a 1:5,000 dilution as a secondary antibody. The tubulin loading control was visualized using mouse 12G10 anti-alpha-tubulin (DSHB) as a primary antibody in a dilution of 1:5000 and horse radish peroxidase-conjugated anti-mouse IgG in a 1:5,000 dilution (Cell Signaling Technology) as a secondary antibody. BSA-MetO protein was used as a positive control. Bands were quantified using ImageJ densitometry analysis.

### Immunofluorescence

For lipid droplet analysis of *Drosophila* intestines or fat bodies, infected flies were dissected, fixed in 4% formaldehyde, washed 3 times in PBS supplemented with 0.1% tween 20 (PBT), and stained with 1 μg/ml DAPI (Sigma) and 2 μg/ml Nile Red (Sigma). Human cells were incubated with the indicated supplements for 3 days, fixed with 4% paraformaldehyde, incubated with BODIPY 493/503 (Thermo Fisher Scientific) and DAPI for 30 mins, washed with PBS, and mounted on slides for confocal microscopy. Lipid droplet number and size were counted using the ImageJ particle analyzer. For phospho-Histone H3 (PH3^+^) staining, guts were incubated first with a polyclonal rabbit anti-phospho-Histone H3 (Ser10) antibody (EMD Millipore) diluted in a ratio of 1:500 in PBT supplemented with 2% BSA. Alexa Fluor 594-conjugated goat anti-rabbit IgG (H+L) antibodies (Thermo Fisher Scientific) were used in a 1:200 ratio to visualize PH3^+^ cells. Samples were then mounted in Vectashield mounting media (Vector Lab Inc) and imaged using an LSM700 confocal microscope (Zeiss).

### Preparation and LC/MS-based metabolomic analysis of samples

Extraction of metabolites followed a previously published protocol [15]. Briefly, to prepare samples for metabolomics, bacteria were cultured overnight in LB broth. For supernatants, 1 ml of a bacterial culture was collected by centrifugation, filtered through 0.22 μm filter (Thermo Fisher Scientific) to remove remaining bacteria, and then combined with methanol to yield a methanol:water solution (80:20). For *Drosophila* studies, the intestines of twenty flies treated as indicated and derived from three independent vials were dissected and homogenized in 500 μl of a cold methanol:water solution (80:20). After incubation for 2h at -80°C, samples were centrifuged for 10 min at 14,000 X g. The supernatants were transferred to a new vial, and the pellets were again extracted with a methanol:water solution (400 μl) as described above. Supernatants from the two extractions were combined. Cecal fluid was prepared by centrifugation for 10 min at 5,000 X g to remove particulates, cells, and bacteria. Methanol was added to yield a methanol:water solution (80:20). This solution was incubated for 6h at -80°C and then centrifuged for 10 min at 14,000 X g. All methanol:water suspensions were lyophilized or dessicated at ambient temperature in a SpeedVac concentrator (Savant). The resulting suspensions were stored at -80°C until use. Metabolite pellets were resuspended in 20 μL LC/MS grade water, and 5 μL were injected over a 15 min gradient using a 5500 QTRAP triple quadrupole mass spectrometer (AB/SCIEX) coupled to a Prominence UFLC HPLC system (Shimadzu) via SRM of a total of 287 SRM transitions using positive and negative polarity switching corresponding to 258 unique endogenous water soluble metabolites. The dwell time was 3 ms per SRM resulting in ∼10–14 data points acquired per detected metabolite. Samples were separated using a Amide XBridge HPLC hydrophilic interaction liquid chromatographic (HILIC) column (3.5 μm; 4.6 mm inner diameter (i.d.) × 100 mm length; Waters) at 300 μl/min. Gradients were run starting from 85% buffer B (HPLC grade acetonitrile) to 40% B from 0–5 min; 40% B to 0% B from 5–16 min; 0% B was held from 16–24 min; 0% B to 85% B from 24–25 min; 85% B was held for 7 min to re-equilibrate the column. Buffer A was comprised of 20 mM ammonium hydroxide/20 mM ammonium acetate (pH = 9.0) in 95:5 water/acetonitrile. Peak areas from the total ion current for each metabolite SRM transition were integrated using MultiQuant version 2.1 software (AB/SCIEX) via the MQ4 peak integration algorithm using a minimum of 8 data points with a 20 sec retention time window.

### Preparation and LC/MS-based lipidomic analysis of samples

The Folch method was used for extraction of lipids [52]. Briefly, the intestines of twenty female flies treated as indicated and harvested from three independent vials were removed and homogenized in a chloroform:methanol solution (2:1, 500 μl). After shaking for 30 minutes, 100 μl of a 0.9% NaCl solution were added, and the mixture was centrifuged for 5 min at 2, 000 rpm to separate the aqueous and organic phases. The lower aqueous phase was dessicated at ambient temperature using a SpeedVac concentrator (Savant). All samples were stored at -80°C prior to LC-MS/MS analysis.

Lipid samples were analyzed as previously described [53]. Briefly, samples were re-suspended in 35 μL of 50% isopropanol (IPA)/50% MeOH. 10 μL of sample were injected onto liquid chromatography tandem mass spectrometry (LC-MS/MS) system. A Cadenza 150 mm x 2 mm 3μm C_18_ column (Imtakt) heated to 40°C at 260 μL/min was used with a 1100 quaternary pump HPLC with room temperature autosampler (Agilent). Lipids were eluted over a 20 min gradient from 32% B buffer (90% IPA/10% ACN/10 mM ammonium formate/0.1% formic acid) to 97% B. A buffer consisted of 59.9% ACN/40% water/10 mM ammonium formate/0.1% formic acid. Lipids were analyzed using a hybrid QExactive Plus Orbitrap mass spectrometer (Thermo Fisher Scientific) in DDA mode using positive/negative ion polarity switching with 1 MS1 scan followed by 8 MS2 HCD scans per cycle (Top 8). DDA data were acquired from m/z 225–1450 in MS1 mode and the resolution was set to 70,000 for MS1 and 35,000 for MS2. MS1 and MS2 target values were set to 5e5 and 1e6, respectively. Lipidomics data were analyzed using LipidSearch 4.1.9 software (Thermo Fisher Scientific). The software identifies intact lipid molecules based on their molecular weight and fragmentation pattern using an internal library of predicted fragment ions per lipid class and the spectra are then aligned based on retention time and MS1 peak areas are quantified across sample conditions.

### Quantification and statistical analysis

All bar graphs represent the mean of at least three biological replicates. Metabolomics experiments were performed in triplicate. For survival curves, thirty female flies were used. Graphpad prism 2.0 software was used to calculate means of pooled data and statistical significance for small data sets and survival curves. A student’s t-test was used to determine the significance of differences between two measurements. The significance of differences in survival curves was calculated using log-rank analysis. The p values for survival curves are shown in the respective graphs. For other data, statistical significance is indicated by stars placed above the compared values, which are defined in the legend. For analysis of metabolomics data, MetaboAnalyst software was used [54]. Data sets were normalized to sum. Means were calculated for each metabolite based on three values, and statistically significant means were assessed using a student’s t-test. In all cases, a p value of less than 0.05 was considered statistically significant.

### Ethics statement

Animal experiments were performed in accordance with standards outlined in the National Research Council’s Guide for the Care and Use of Laboratory Animals and Boston Childrens Hospital’s public health service Assurance. The protocol was approved by the Boston Children’s Hospital Institutional Animal Care and Use Committee (IACUC) appointed to review proposals for research involving vertebrate animals (Protocol number 14-06-2706). For euthanasia, rabbits were anesthetized with ketamine/xylazine followed by administration of fatal plus. All efforts were made to minimize distress, pain, and suffering.

## Supporting information

S1 Fig Provision of *gcvT* in trans rescues the virulence of a *V. cholerae* Δ*gcvT* mutant. Survival curves of flies fed LB broth inoculated with wild-type *V*. *cholerae* (WT) or a ΔgcvT mutant harboring a pBAD plasmid expressing *lacZ* (pBAD-*lacZ*) or *gcvT* (pBAD-*gcvT*). Statistical significance was calculated by log-rank analysis.
(PDF)

S2 Fig The host intestine senses glycine released by the Δ*gcvT* mutant, but glycine ingestion does not prolong host survival. (A-D) qRT-PCR measurements of *Drosophila* (A) *gcvH*, (B) *gcvL*, (C) *gcvT* and (D) *gcvP* genes in the intestines of flies fed LB alone or inoculated with wild-type *V*. *cholerae* (WT) or a Δ*gcvT* mutant. (E) Survival curves of flies fed LB broth inoculated with wild-type *V*. *cholerae* supplemented concentrations of glycine as noted. (F) Enumeration of PH3^+^ positive cells in the intestines of flies fed with PBS or PBS supplemented with 50mM glycine (Gly) at 72h. For pairwise comparisons, a p-value was calculated using the Student’s t test (*p<0.05, **p<0.01).
(PDF)

S3 Fig Mutation of the *V. cholerae* glycine cleavage system alters the extracellular environment in the *Drosophila* intestine. LC-MS/MS based metabolomic comparison of the intestines of *Drosophila* fed LB broth, wild-type *V*. *cholerae* (WT) or a Δ*gcvT* mutant. Error bars represent the standard deviation of experimental triplicates.
(PDF)

S4 Fig A *V. cholerae* glycine cleavage system mutant does not have a colonization defect in the infant rabbit intestine. Bacterial burden in the terminal ileum (A) and cecum (B) of infant rabbits infected with wild-type *V*. *cholerae* (WT) or a Δ*gcvT* mutant. (C) Volume of cecal fluid harvested from the intestines of infant rabbits infected with wild-type *V*. *cholerae* (WT) or a Δ*gcvT* mutant.
(PDF)

S5 Fig MetO but not other metabolites differentially elevated in a *V. cholerae* Δ*gcvT* infection increases host survival and normalizes intestinal lipid mobilization and insulin signaling. (A) Fractional survival of flies fed LB broth inoculated with wild-type *V*. *cholerae* (WT) and supplemented with the indicated metabolites. (B) Growth curves of wild-type *V*. *cholerae* in LB supplemented with different concentrations of MetO. (C) Fractional survival of flies fed LB broth inoculated with 50mM methionine (Met) or methionine sulfoxide (MetO). (D) Growth curves of wild-type *V*. *cholerae* in LB supplemented with various concentrations of methionine. (E) Western blot analysis of phosphorylated AKT or total AKT levels in whole flies fed LB broth supplemented with 50mM methionine (Met) or 50mM methionine sulfoxide (MetO). (F) Nile red staining of neutral lipids in the fat body and intestine of flies fed LB broth supplemented with 50mM methionine (Met) or 50mM methionine sulfoxide (MetO). (G) Quantification of cells with lipid droplets in midgut of flies treated as in (F). (H) Bodipy staining of neutral lipids in HEK93 cells incubated with PBS or 100mM methionine sulfoxide (MetO). (I) Number of lipid droplets per positive cell when incubated with PBS or 100mM methionine sulfoxide (MetO). For pairwise comparisons, a p-value was calculated using the Student’s t test (***p<0.001).
(PDF)

S6 Fig LC-MS/MS based lipidomic analysis of the intestines of flies fed with LB broth alone (LB) or inoculated with wild-type *V. cholerae* (Vc). (A) Complete analysis of lipid subgroups. (B) Lipid distribution by chain length (peak threshold = 1), * denotes statistical significance calculated using a student’s t-test. PC: phosphatidylcholine, PE: phosphatidylethanolamine, TG: triglyceride, DG: diglyceride, dMePE: dimethylphosphatidylethanolamine, CL: cardiolipin, Cer: ceramide, LPI: lysophosphatidylinositol, LPS: lysophosphatidylserine, PA: phosphatidic acid, MG: monoglyceride, MGDG: monogalactosyldiacylglycerol, Pet: phosphatidylethanol, PS: phosphatidylserine, PG: phosphatidylglycerol, PI: phosphatidylinositol, PIP: phosphatidylinositol phosphate, SM: sphingomyelin, So: sphingosine.
(PDF)

S7 Fig LC-MS/MS based lipidomic analysis of the intestines of flies infected with wild-type (WT) or Δ*gcvT* mutant *V. cholerae*. (A) Complete analysis of lipid subgroups. (B) Lipid distribution by chain length (minimum threshold = 1), * denotes statistical significance calculated using a student’s t-test. PC: phosphatidylcholine, PE: phosphatidylethanolamine, TG: triglyceride, DG: diglyceride, dMePE: dimethylphosphatidylethanolamine, CL: cardiolipin, Cer: ceramides, LPI: lysophosphatidylinositol, LPS: lysophosphatidylserine, PA: phosphatidic acid, MG: monoglyceride, MGDG: monogalactosyldiacylglycerol, Pet: phosphatidylethanol, PS: phosphatidylserine, PG: phosphatidylglycerol, PI: phosphatidylinositol, PIP: phosphatidylinositol phosphate, SM: sphingomyelin, So: sphingosine
(PDF)

S8 Fig LC-MS/MS based lipidomics analysis of the intestines of flies infected with wild-type V. *cholerae* (WT) or a Δ*acs1* mutant. (A) Complete analysis of lipid subgroups. (B) Lipid distribution by chain length (peak threshold = 1), * denotes statistical significance calculated using a student’s t-test. PC: phosphatidylcholine, PE: phosphatidylethanolamine, TG: triglyceride, DG: diglyceride, dMePE: dimethylphosphatidylethanolamine, CL: Cardiolipin, Cer: Ceramides, LPI: lysophosphatidylinositol, LPS: lysophosphatidylserine, PA: phosphatidic acid, MG: monoglyceride, MGDG: Monogalactosyldiacylglycerol, Pet: phosphatidylethanol, PS: phosphatidylserine, PG: phosphatidylglycerol, PI: phosphatidylinositol, PIPI: phosphatidylinositol, SM: sphingomyelin, So: Sphingoshine
(PDF)

S9 Fig LC-MS/MS based lipidomic analysis of the intestines of *msrA*^EY05753^ mutant flies fed LB broth (LB) or infected with wild-type *V. cholerae* (Vc). (A) Complete analysis of lipid subgroups. (B) Lipid distribution by chain length (peak threshold = 1), * denotes statistical significance calculated using a student’s t-test. PC: phosphatidylcholine, PE: phosphatidylethanolamine, TG: triglyceride, DG: diglyceride, dMePE: dimethylphosphatidylethanolamine, CL: Cardiolipin, Cer: Ceramides, LPI: lysophosphatidylinositol, LPS: lysophosphatidylserine, PA: phosphatidic acid, MG: monoglyceride, MGDG: Monogalactosyldiacylglycerol, Pet: phosphatidylethanol, PS: phosphatidylserine, PG: phosphatidylglycerol, PI: phosphatidylinositol, PIPI: phosphatidylinositol, SM: sphingomyelin, So: Sphingoshine.
(PDF)

S1 Table Metabolomic analysis of LB broth and the spent supernatants of wild-type *V. cholera*e or a Δ*gcvT* mutant cultured in LB broth (normalized by sum). (XLSX)

S2 Table Metabolomic analysis of the intestines of flies fed LB broth alone or inoculated with wild-type *V. cholerae* or a Δ*gcvT* mutant (normalized by sum). (XLSX)

S3 Table Metabolomic analysis of the cecal fluid of rabbits infected with wild-type *V. cholerae* or a Δ*gcvT* mutant (normalized by sum). (XLSX)

S4 Table Lipidomic analysis of the intestines of flies fed with LB broth alone or inoculated with wild-type *V. cholerae* or a Δ*acs1* mutant (normalized by sum). (XLSX)

S5 Table Lipidomic analysis of the intestines of flies fed with LB broth inoculated with wild-type *V. cholerae* or a Δ*gcvT* mutant (normalized by sum). (XLSX)

S6 Table Lipidomic analysis of the intestines of *msrA*^EY05753^ mutant flies fed with LB broth alone or inoculated with wild-type *V. cholerae* (normalized by sum). (XLSX)

S7 Table Fly strains. (PDF)

S8 Table Strains, plasmids and oligonucleotides. (PDF)

S9 Table Oligonucleotides for qPCR. (PDF)

S1 References Supporting references. (PDF)
