# Supplementary figures and images for: Vibrio cholerae ensures function of host proteins required for virulence through consumption of luminal methionine sulfoxide
Source: PLoS Pathog. 2017 Jun 6;13(6):e1006428. doi: 10.1371/journal.ppat.1006428 (PMC5473594; doi:10.1371/journal.ppat.1006428)

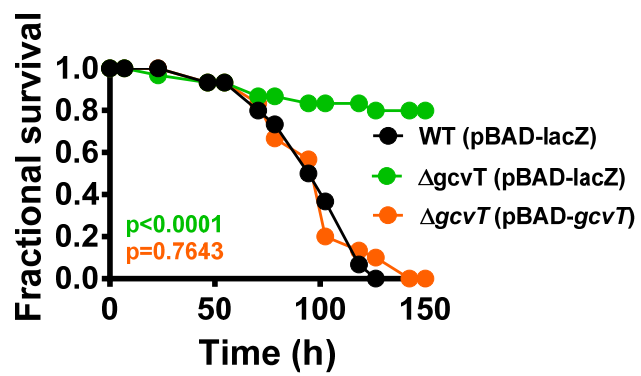

Supplement: S1 Fig — Survival curves of flies fed LB broth inoculated with wild-type V. cholerae (WT) or a ΔgcvT mutant harboring a pBAD plasmid expressing lacZ (pBAD-lacZ) or gcvT (pBAD-gcvT). Statistical significance was calculated by log-rank analysis. (PDF) [file ppat.1006428.s001.pdf]

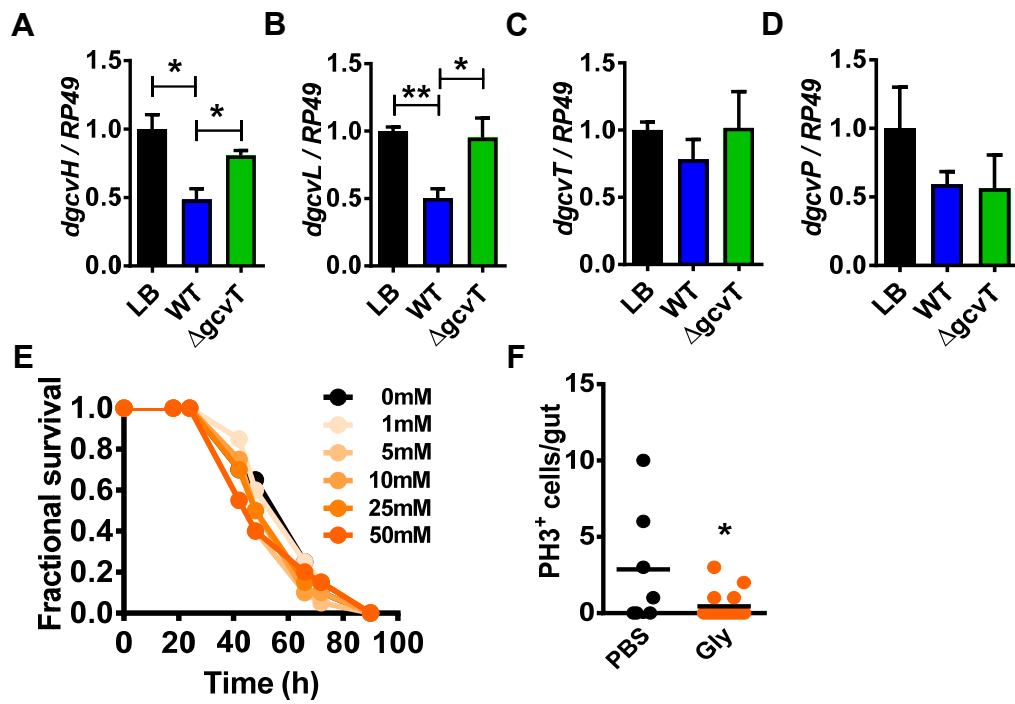

Supplement: S2 Fig — (A-D) qRT-PCR measurements of Drosophila (A) gcvH, (B) gcvL, (C) gcvT and (D) gcvP genes in the intestines of flies fed LB alone or inoculated with wild-type V. cholerae (WT) or a ΔgcvT mutant. (E) Survival curves of flies fed LB broth inoculated with wild-type V. cholerae supplemented concentrations of glycine as noted. (F) Enumeration of PH3+ positive cells in the intestines of flies fed with PBS or PBS supplemented with 50mM glycine (Gly) at 72h. For pairwise comparisons, a p-value was calculated using the Student’s t test (*p<0.05, **p<0.01). (PDF) [file ppat.1006428.s002.pdf]

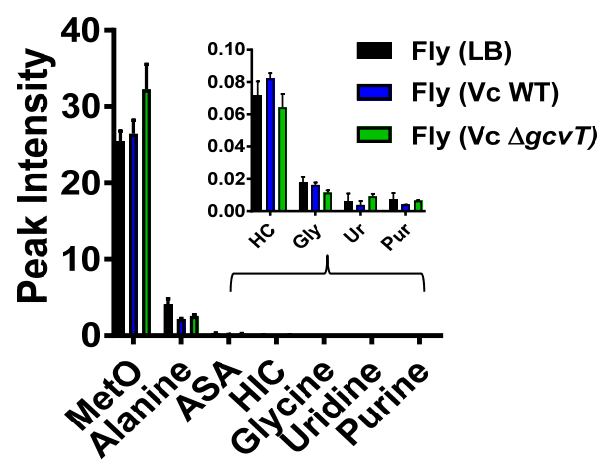

Supplement: S3 Fig — LC-MS/MS based metabolomic comparison of the intestines of Drosophila fed LB broth, wild-type V. cholerae (WT) or a ΔgcvT mutant. Error bars represent the standard deviation of experimental triplicates. (PDF) [file ppat.1006428.s003.pdf]

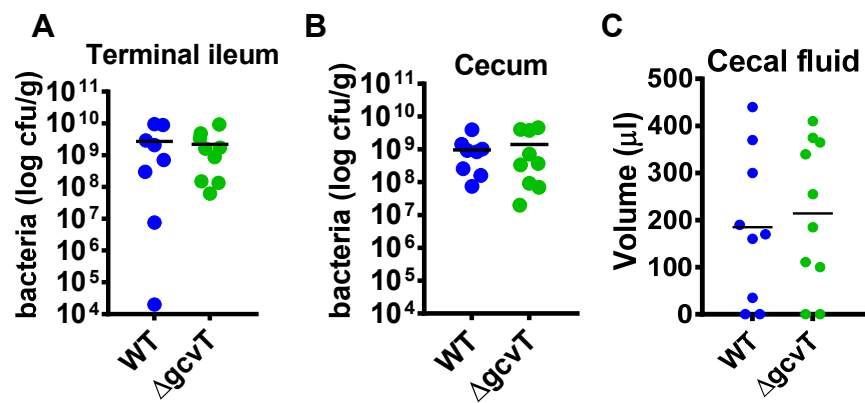

Supplement: S4 Fig — Bacterial burden in the terminal ileum (A) and cecum (B) of infant rabbits infected with wild-type V. cholerae (WT) or a ΔgcvT mutant. (C) Volume of cecal fluid harvested from the intestines of infant rabbits infected with wild-type V. cholerae (WT) or a ΔgcvT mutant. (PDF) [file ppat.1006428.s004.pdf]

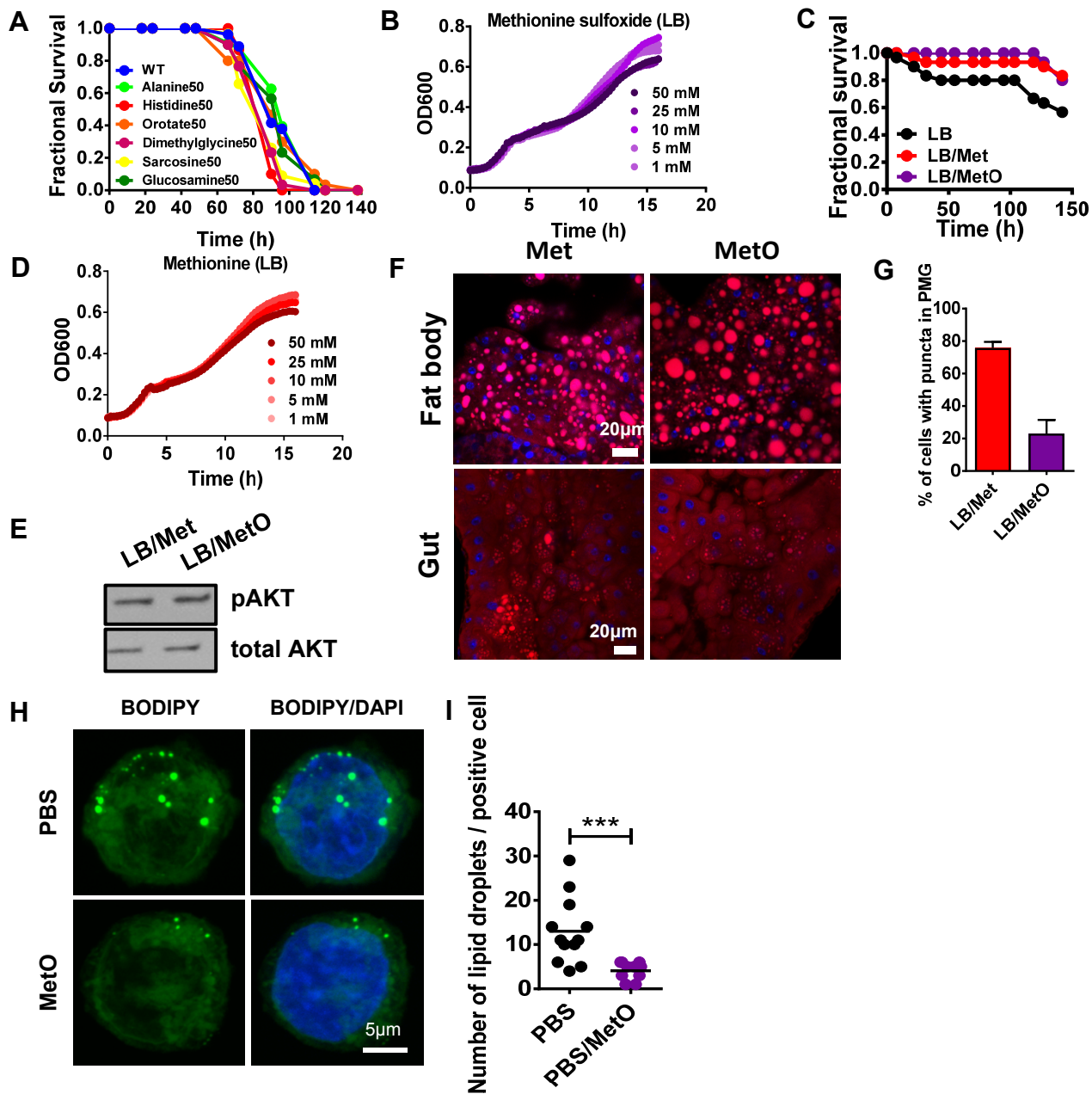

Supplement: S5 Fig — (A) Fractional survival of flies fed LB broth inoculated with wild-type V. cholerae (WT) and supplemented with the indicated metabolites. (B) Growth curves of wild-type V. cholerae in LB supplemented with different concentrations of MetO. (C) Fractional survival of flies fed LB broth inoculated with 50mM methionine (Met) or methionine sulfoxide (MetO). (D) Growth curves of wild-type V. cholerae in LB supplemented with various concentrations of methionine. (E) Western blot analysis of phosphorylated AKT or total AKT levels in whole flies fed LB broth supplemented with 50mM methionine (Met) or 50mM methionine sulfoxide (MetO). (F) Nile red staining of neutral lipids in the fat body and intestine of flies fed LB broth supplemented with 50mM methionine (Met) or 50mM methionine sulfoxide (MetO). (G) Quantification of cells with lipid droplets in midgut of flies treated as in (F). (H) Bodipy staining of neutral lipids in HEK93 cells incubated with PBS or 100mM methionine sulfoxide (MetO). (I) Number of lipid droplets per positive cell when incubated with PBS or 100mM methionine sulfoxide (MetO). For pairwise comparisons, a p-value was calculated using the Student’s t test (***p<0.001). (PDF) [file ppat.1006428.s005.pdf]

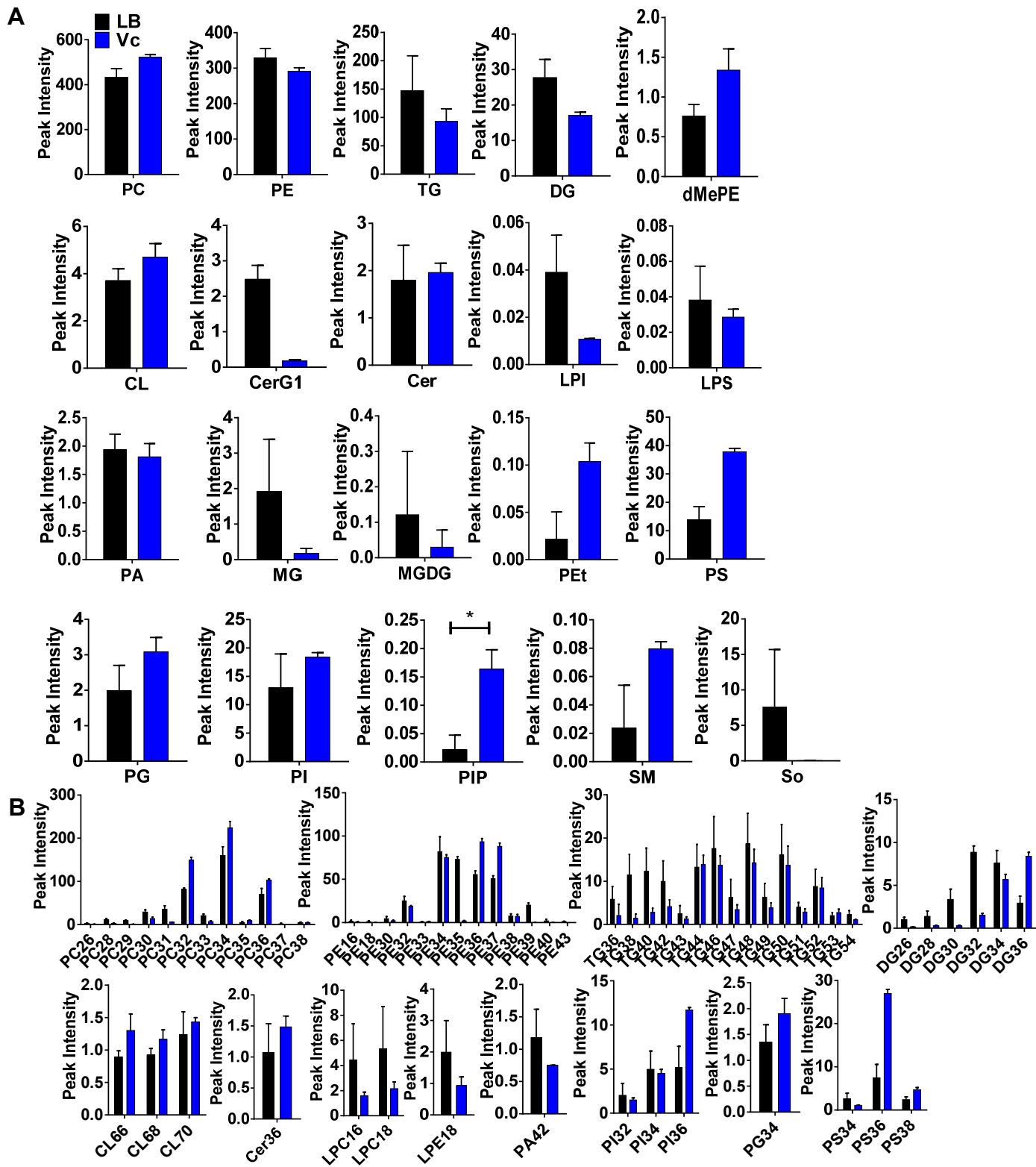

Supplement: S6 Fig — (A) Complete analysis of lipid subgroups. (B) Lipid distribution by chain length (peak threshold = 1), * denotes statistical significance calculated using a student’s t-test. PC: phosphatidylcholine, PE: phosphatidylethanolamine, TG: triglyceride, DG: diglyceride, dMePE: dimethylphosphatidylethanolamine, CL: cardiolipin, Cer: ceramide, LPI: lysophosphatidylinositol, LPS: lysophosphatidylserine, PA: phosphatidic acid, MG: monoglyceride, MGDG: monogalactosyldiacylglycerol, Pet: phosphatidylethanol, PS: phosphatidylserine, PG: phosphatidylglycerol, PI: phosphatidylinositol, PIP: phosphatidylinositol phosphate, SM: sphingomyelin, So: sphingosine. (PDF) [file ppat.1006428.s006.pdf]

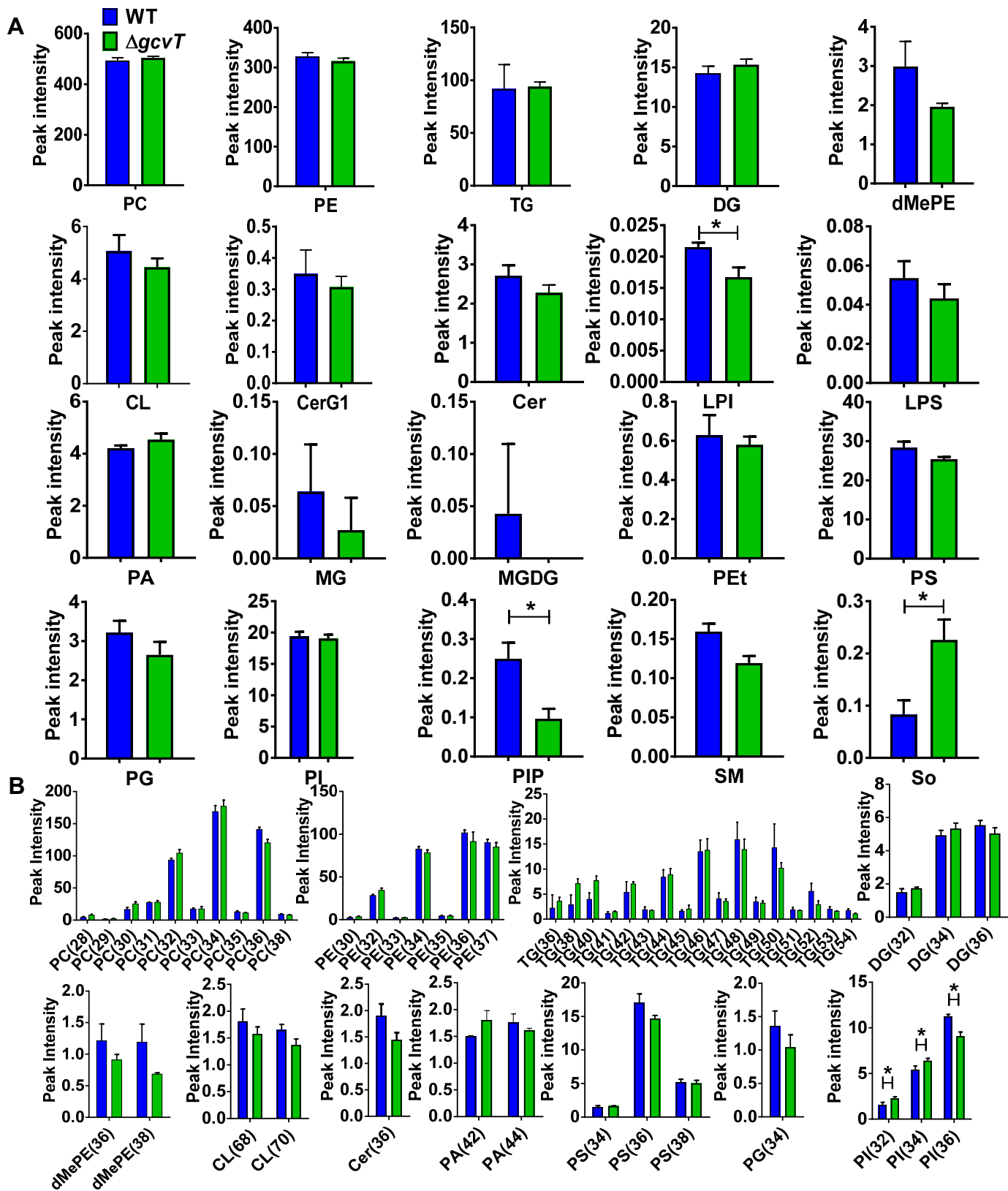

Supplement: S7 Fig — (A) Complete analysis of lipid subgroups. (B) Lipid distribution by chain length (minimum threshold = 1), * denotes statistical significance calculated using a student’s t-test. PC: phosphatidylcholine, PE: phosphatidylethanolamine, TG: triglyceride, DG: diglyceride, dMePE: dimethylphosphatidylethanolamine, CL: cardiolipin, Cer: ceramides, LPI: lysophosphatidylinositol, LPS: lysophosphatidylserine, PA: phosphatidic acid, MG: monoglyceride, MGDG: monogalactosyldiacylglycerol, Pet: phosphatidylethanol, PS: phosphatidylserine, PG: phosphatidylglycerol, PI: phosphatidylinositol, PIP: phosphatidylinositol phosphate, SM: sphingomyelin, So: sphingosine (PDF) [file ppat.1006428.s007.pdf]

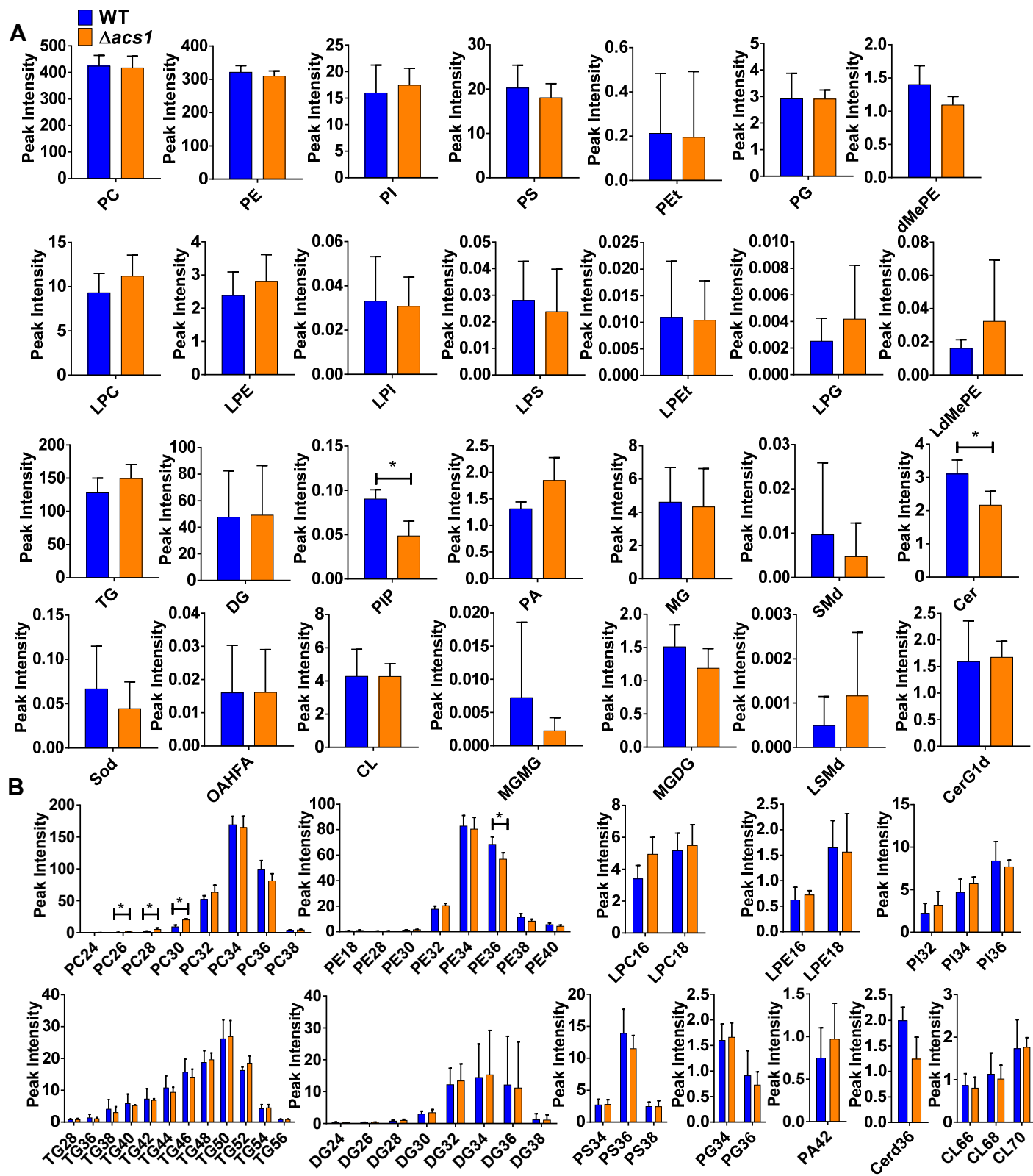

Supplement: S8 Fig — (A) Complete analysis of lipid subgroups. (B) Lipid distribution by chain length (peak threshold = 1), * denotes statistical significance calculated using a student’s t-test. PC: phosphatidylcholine, PE: phosphatidylethanolamine, TG: triglyceride, DG: diglyceride, dMePE: dimethylphosphatidylethanolamine, CL: Cardiolipin, Cer: Ceramides, LPI: lysophosphatidylinositol, LPS: lysophosphatidylserine, PA: phosphatidic acid, MG: monoglyceride, MGDG: Monogalactosyldiacylglycerol, Pet: phosphatidylethanol, PS: phosphatidylserine, PG: phosphatidylglycerol, PI: phosphatidylinositol, PIPI: phosphatidylinositol, SM: sphingomyelin, So: Sphingoshine (PDF) [file ppat.1006428.s008.pdf]

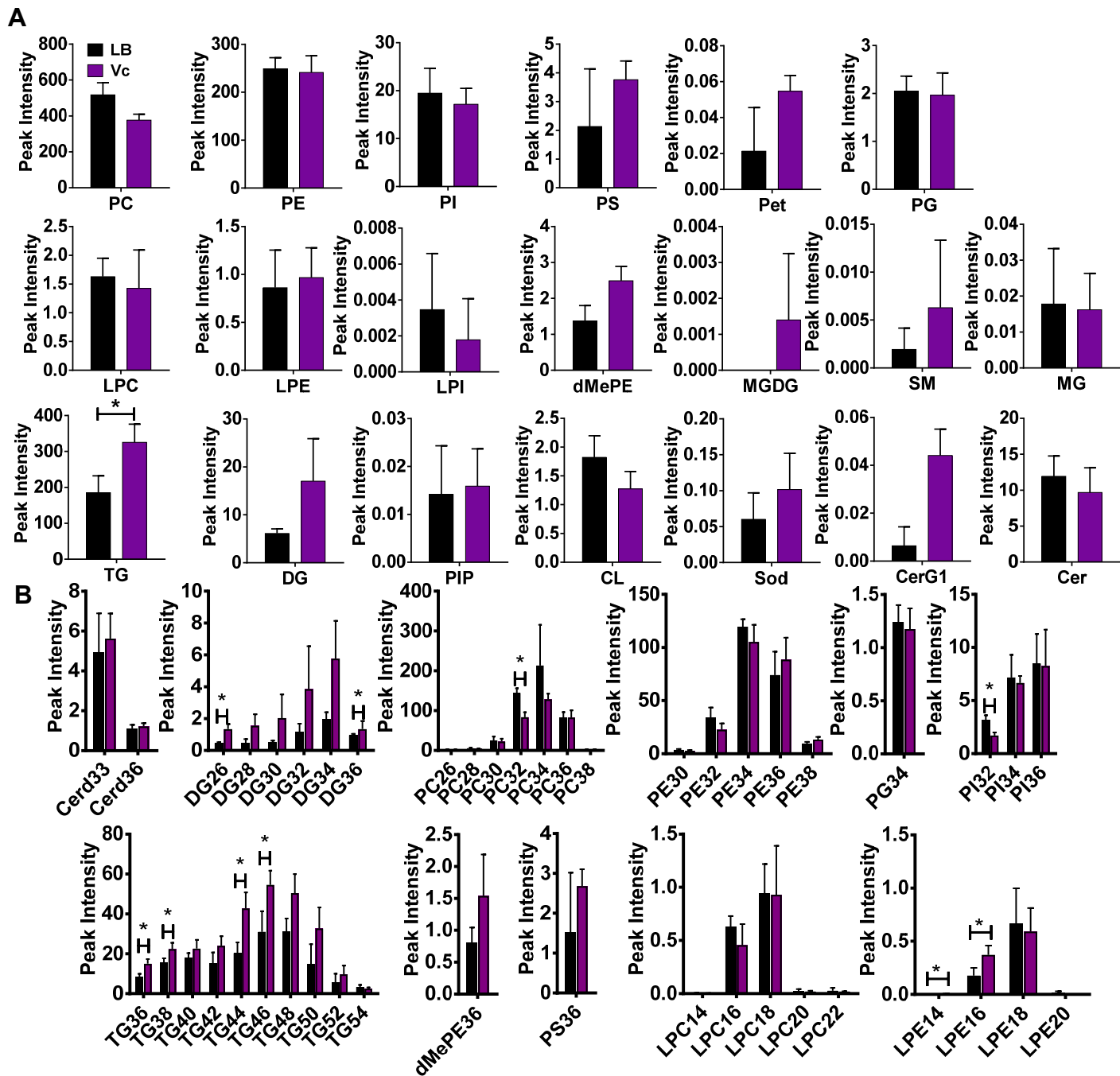

Supplement: S9 Fig — (A) Complete analysis of lipid subgroups. (B) Lipid distribution by chain length (peak threshold = 1), * denotes statistical significance calculated using a student’s t-test. PC: phosphatidylcholine, PE: phosphatidylethanolamine, TG: triglyceride, DG: diglyceride, dMePE: dimethylphosphatidylethanolamine, CL: Cardiolipin, Cer: Ceramides, LPI: lysophosphatidylinositol, LPS: lysophosphatidylserine, PA: phosphatidic acid, MG: monoglyceride, MGDG: Monogalactosyldiacylglycerol, Pet: phosphatidylethanol, PS: phosphatidylserine, PG: phosphatidylglycerol, PI: phosphatidylinositol, PIPI: phosphatidylinositol, SM: sphingomyelin, So: Sphingoshine. (PDF) [file ppat.1006428.s009.pdf]
