# Supplementary material for: Vibrio cholerae ensures function of host proteins required for virulence through consumption of luminal methionine sulfoxide
Source: PLoS Pathog. 2017 Jun 6;13(6):e1006428. doi: 10.1371/journal.ppat.1006428 (PMC5473594; doi:10.1371/journal.ppat.1006428)
Supplement: S7 Table — (PDF) [file ppat.1006428.s016.pdf]

S7 Table. Fly strains.

Description	Genotype	Gene	Reference	Flybase ID
Wild type strains				
OregonR			Laboratory stock	
yw	y1w1		BDRC (1495)	FBst0001495
Transposon insertion mutant strains				
$MsrA^{EY05753}$	y[1] w[67c23]; P{w[+mC] y[+mDint2]=EPgy2}Eip71CD[EY05753]	CG7266	BDRC (16671)	FBst0016671
$MsrA^{MI14018}$	y[1] w[*]; Mi{y[+mDint2]=MIC}Eip71CD[MI14018]	CG7266	BDRC (59248)	FBst0059248
UAS-RNAi strain				
$msrA^{RNAi}$	w[1118]; P{GD17043}v48990/TM3	CG7266	VDRC (v48990)	FBst0468225
Drivers				
NP1-Gal4	NP1-Gal4 (II)		[1]	
Da-Gal4	w[1118]; P{da-GAL4.w[-]}3		[2]	
Act-Gal4	yw; act5cGal4/TM6B, Tb		[3]	

BDRC: Bloomington Drosophila Stock Center

VDRC: Vienna Drosophila Resource Center
