# Supplementary material for: Vibrio cholerae ensures function of host proteins required for virulence through consumption of luminal methionine sulfoxide
Source: PLoS Pathog. 2017 Jun 6;13(6):e1006428. doi: 10.1371/journal.ppat.1006428 (PMC5473594; doi:10.1371/journal.ppat.1006428)
Supplement: S8 Table — (PDF) [file ppat.1006428.s017.pdf]

S8 Table. Strains, plasmids and oligonucleotides.

Description/sequence		Reference
Bacterial strains		
PW724	*V. cholerae* MO10; Sm^r	[4]
PW1640	MO10 Δ*gcvT* ; Sm^r	This study
PW1658	MO10 Δ*gcvP* ; Sm^r	This study
PW1695	MO10 ΔVC2638; Sm^r	This study
PW1698	MO10 Δ*gcvH* ; Sm^r	This study
PW1692	MO10 Δ*glyA* 1Δ*glyA2* ; Sm^r	This study
PW1405	MO10 Δ*acs1* ; Sm^r	[5]
SM10λpir	*E. coli* thi thr leu tonA lacY supE recA::RP4-2-Tc::Mu (λpirR6K) Km^r	[6]
Plasmids		
pWM91	oriR6K mobRP4 *lacI* pTac *tnp* mini-Tn*10* ; Km^r Ap^r	[7]
pWM91::Δ*gcvT*	pWM91 carrying an unmarked, in-frame deletion in VCA0280 (gcvT); Ap^r	This study
pWM91::Δ*gcvP*	pWM91 carrying an unmarked, in-frame deletion in VCA0276 (gcvP); Ap^r	This study
pWM91::ΔVC2638	pWM91 carrying an unmarked, in-frame deletion in VC2638; Ap^r	This study
pWM91::Δ*gcvH*	pWM91 carrying an unmarked, in-frame deletion in VCA0277 (gcvH); Ap^r	This study
pWM91::Δ*glyA1*Δ*glyA2*	pWM91 carrying an unmarked, in-frame deletion in VCA0941/VC0278 (glyA1/glyA2); Ap^r	This study
pBAD-TOPO	arabinose inducible expression vector used to generate His6/V5 epitope tagged proteins; Ap^r	Life Technologies
pBAD-TOPO-*lacZ*	pBAD-TOPO control vector containing *lacZ* gene; Ap^r	Life Technologies
pBAD-TOPO-*gcvT*	pBAD-TOPO containing *gcvT* gene; Ap^r	This study
Oligonucleotides		
Mutagenesis		
ΔVCA0280		
gcvT#1	5'-CCGCTCGAGGCGAATTTTATGAGCCGGTA-3'	This study
gcvT#2	5'-TAACGAGCGGCCGCATAGCGGTGTCGTCAGTAAGGTT-3'	This study
gcvT#3	5'-TGCGGCCGCTCGTTAGGGAAGATGCTGCCAATGACT-3'	This study
gcvT#4	5'-GGACTAGTGGACTTTTTGTGGAGGTTGG-3'	This study
ΔVCA0276		
gcvP#1	5'-CCGCTCGAGCACTGGTTGAGTCGGTGAAA-3'	This study
gcvP#2	5'-TAACGAGCGGCCGCAGCGTGCAACGAACTCATTTTG-3'	This study
gcvP#3	5'-TGCGGCCGCTCGTTAAAATACTGGCCAACGGTGAAC-3'	This study
gcvP#4	5'-GGACTAGTTACTTTGGGCGGAATTATCG-3'	This study
ΔVC2638		
VC2638#1	5'-CCGCTCGAGTGTTTATCGAACCGAATGAGC-3'	This study
VC2638#2	5'-TAACGAGCGGCCGCAGGCGACATCAACGTGAATCTG-3'	This study
VC2638#3	5'-TGCGGCCGCTCGTTAGGCCCTGAGATGATCAAAC-3'	This study
VC2638#4	5'-GGACTAGTTAAAGCCCCAAAACCAAATG-3'	This study
ΔVCA0277		
gcvH#1	5'-CCGCTCGAGCGGATACGCATCTCATGTTG-3'	This study
gcvH#2	5'-TAACGAGCGGCCGCAAACCCATTCATGGCTTTCTG-3'	This study
gcvH#3	5'-TGCGGCCGCTCGTTAAAAGACGCGGAAGAGTACC-3'	This study
gcvH#4	5'-GGACTAGTTTCATCGTTGCCAGCATATC-3'	This study
ΔVC0941		
glyA1#1	5'-CCGCTCGAGCAGAATCGCGATCCAATCTT-3'	This study
glyA1#2	5'-TAACGAGCGGCCGCATTCCTGAATGGCAGCGTAT-3'	This study
glyA1#3	5'-TGCGGCCGCTCGTTAGTGATTGAAGCGACGAAAC-3'	This study
glyA1#4	5'-GGACTAGTATGATGGCCAAGTACGCATT-3'	This study
ΔVCA0278		
glyA2#1	5'-CCGCTCGAGATTGTGGCATGAAGCGATTT-3'	This study
glyA2#2	5'-TAACGAGCGGCCGCACGCCAGTGGGGTAGAGAAGAA-3'	This study
glyA2#3	5'-TGCGGCCGCTCGTTAGACGTTGAGCAACAAGTGC-3'	This study
glyA2#4	5'-GGACTAGTCGGACATTTTCACTTTCACG-3'	This study
Complementation		
gcvT-Fc	5'-GAGGAATAATAAATGACTGAACAACACGAAAC-3'	This study
gcvT-Rc	5'-TGCTCCGCGATAATAACGCT-3'	This study
