# Supplementary material for: Vibrio cholerae ensures function of host proteins required for virulence through consumption of luminal methionine sulfoxide
Source: PLoS Pathog. 2017 Jun 6;13(6):e1006428. doi: 10.1371/journal.ppat.1006428 (PMC5473594; doi:10.1371/journal.ppat.1006428)
Supplement: S9 Table — (PDF) [file ppat.1006428.s018.pdf]

S9 Table. Oligonucleotides for qPCR

Primers	Sequence	Description	Reference
dmsrA-Fw	5'-CAAGGATCTGAGCACCGTTCG-3'	Ecdysone-induced protein 28/29kD (Eip71CD) - CG7266	[8]
dmsrA-Rv	5'-GGTACGTTGGCAAATCCGAGC-3'		
dgcvH-Fw	5'-CGGTTGGAAAATTCATCTCG-3'	pumpless (gcvH) - CG7758	This study
dgcvH-Rv	5'-CCCACTCGTGTTTGTTTGTG-3'		
dgcvT-Fw	5'-CCAGGGAAAAGACGTATCCA-3'	glycine cleavage system T (gcvT) - CG6415	This study
dgcvT-Rv	5'-CGGTGTAACCACATCTCGTG-3'		
dgcvP-Fw	5'-GATGATCGAGCCCACTGAAT-3'	glycine cleavage system P (gcvP) - CG3999	This study
dgcvP-Rv	5'-AGCCTGCTCCCGTGTATATG-3'		
dgcvL-Fw	5'-CTTTGGCAAACCCATCAACT-3'	glycine cleavage system P (gcvL) - CG7430	This study
dgcvL-Rv	5'-CTGGCGCCATTACCATTATT-3'		
RP49-Fw	5'-TACAGGCCCAAGATCGTGAA-3'	Ribosomal protein L32 (rpl32) - CG7939	[9]
RP49-Rv	5'-TCTCCTTGCGCTTCTTGGA-3'		
