# Supplementary material for: Vibrio cholerae ensures function of host proteins required for virulence through consumption of luminal methionine sulfoxide
Source: PLoS Pathog. 2017 Jun 6;13(6):e1006428. doi: 10.1371/journal.ppat.1006428 (PMC5473594; doi:10.1371/journal.ppat.1006428)
Supplement: S1 References — (PDF) [file ppat.1006428.s019.pdf]

S1 References. Supporting references list

1. Morgan NS, Skovronsky DM, Artavanis-Tsakonas S, Mooseker MS. The molecular cloning and characterization of *Drosophila melanogaster* myosin-IA and myosin-IB. J Mol Biol. 1994;239(3):347-56. Epub 1994/06/10. doi: S0022-2836(84)71376-3 [pii]10.1006/jmbi.1994.1376. PubMed PMID: 8201616.

2. Wodarz A, Hinz U, Engelbert M, Knust E. Expression of crumbs confers apical character on plasma membrane domains of ectodermal epithelia of *Drosophila*. Cell. 1995;82(1):67-76. Epub 1995/07/14. doi: 0092-8674(95)90053-5 [pii]. PubMed PMID: 7606787.

3. Ito K, Awano W, Suzuki K, Hiromi Y, Yamamoto D. The *Drosophila* mushroom body is a quadruple structure of clonal units each of which contains a virtually identical set of neurons and glial cells. Development. 1997;124(4):761-71. Epub 1997/02/01. PubMed PMID: 9043058.

4. Waldor MK, Mekalanos JJ. Emergence of a new cholera pandemic: molecular analysis of virulence determinants in *Vibrio cholerae* O139 and development of a live vaccine prototype. J Infect Dis. 1994;170(2):278-83. Epub 1994/08/01. PubMed PMID: 8035010.

5. Hang S, Purdy AE, Robins WP, Wang Z, Mandal M, Chang S, et al. The acetate switch of an intestinal pathogen disrupts host insulin signaling and lipid metabolism. Cell Host Microbe. 2014;16(5):592-604. Epub 2014/12/20. doi: 10.1016/j.chom.2014.10.006 S1931-3128(14)00384-9 [pii]. PubMed PMID: 25525791; PubMed Central PMCID: PMC4272434.

6. Miller VL, Mekalanos JJ. A novel suicide vector and its use in construction of insertion mutations: osmoregulation of outer membrane proteins and virulence determinants in Vibrio cholerae requires toxR. J Bacteriol. 1988;170(6):2575-83. Epub 1988/06/01. PubMed PMID: 2836362; PubMed Central PMCID: PMC211174.

7. Metcalf WW, Jiang W, Daniels LL, Kim SK, Haldimann A, Wanner BL. Conditionally replicative and conjugative plasmids carrying lacZ alpha for cloning, mutagenesis, and allele replacement in bacteria. Plasmid. 1996;35(1):1-13. Epub 1996/01/01. doi: S0147-619X(96)90001-3 [pii] 10.1006/plas.1996.0001. PubMed PMID: 8693022.

8. Chung H, Kim AK, Jung SA, Kim SW, Yu K, Lee JH. The *Drosophila* homolog of methionine sulfoxide reductase A extends lifespan and increases nuclear localization of FOXO. FEBS Lett. 2010;584(16):3609-14. Epub 2010/07/27. doi: 10.1016/j.febslet.2010.07.033S0014-5793(10)00591-0 [pii]. PubMed PMID: 20655917.

9. LaLonde M, Janssens H, Yun S, Crosby J, Redina O, Olive V, et al. A role for Phospholipase D in *Drosophila* embryonic cellularization. BMC Dev Biol. 2006;6:60. Epub

2006/12/13. doi: 1471-213X-6-60 [pii]10.1186/1471-213X-6-60. PubMed PMID: 17156430; PubMed Central PMCID: PMC1698916.
